# PASSpedia: A Polyadenylation Site Database Across Different Species at Single‐cell Resolution

**DOI:** 10.1093/gpbjnl/qzaf089

**Published:** 2025-09-23

**Authors:** Pei-Hong Zhang, Hua Feng, Xu-Kai Ma, Fang Nan, Li Yang

**Affiliations:** Center for Molecular Medicine, Children’s Hospital of Fudan University and Shanghai Key Laboratory of Medical Epigenetics, International Laboratory of Medical Epigenetics and Metabolism, Ministry of Science and Technology, Institutes of Biomedical Sciences, Fudan University, Shanghai 200032, China; Shanghai Institute of Nutrition and Health, University of Chinese Academy of Sciences, Chinese Academy of Sciences, Shanghai 200031, China; Center for Molecular Medicine, Children’s Hospital of Fudan University and Shanghai Key Laboratory of Medical Epigenetics, International Laboratory of Medical Epigenetics and Metabolism, Ministry of Science and Technology, Institutes of Biomedical Sciences, Fudan University, Shanghai 200032, China; Center for Molecular Medicine, Children’s Hospital of Fudan University and Shanghai Key Laboratory of Medical Epigenetics, International Laboratory of Medical Epigenetics and Metabolism, Ministry of Science and Technology, Institutes of Biomedical Sciences, Fudan University, Shanghai 200032, China; Center for Molecular Medicine, Children’s Hospital of Fudan University and Shanghai Key Laboratory of Medical Epigenetics, International Laboratory of Medical Epigenetics and Metabolism, Ministry of Science and Technology, Institutes of Biomedical Sciences, Fudan University, Shanghai 200032, China; Center for Molecular Medicine, Children’s Hospital of Fudan University and Shanghai Key Laboratory of Medical Epigenetics, International Laboratory of Medical Epigenetics and Metabolism, Ministry of Science and Technology, Institutes of Biomedical Sciences, Fudan University, Shanghai 200032, China

**Keywords:** Polyadenylation, Database, Single-cell RNA-seq, Alternative polyadenylation, Multi-species conservation

## Abstract

Polyadenylation site (PAS) selection plays important roles in gene expression regulation and function. RNA sequencing (RNA-seq) data derived from 3′ tag sequencing contain intrinsic information about PAS usage and have been analyzed for alternative polyadenylation (APA) isoform expression in both bulk and single‐cell samples. Here, we upgraded our previously developed deep learning-based PAS analysis pipeline SCAPTURE v2 to profile PASs from 1330 published 3′ tag-based single-cell RNA-seq (scRNA-seq) datasets across seven species, resulting in a comprehensive PAS landscape across species. Validation with long-read sequencing data from matched human tissues showed high accuracy of single-cell PAS profiling by SCAPTURE, including previously unannotated ones. Further comparisons revealed distinct PAS usage preferences in different species, such as human *versus* mouse, independent of conservation of gene expression. Finally, we present PASSpedia, a comprehensive database for PAS analysis and comparison across seven species at single‐cell resolution, which is freely accessible online at https://bits.fudan.edu.cn/PASSpedia/.

## Graphical abstract

**Figure:**
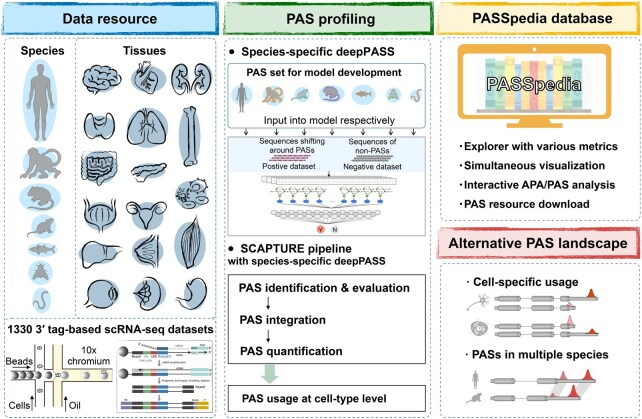

## Introduction

Multiple co- and/or post-transcriptional processes, such as 5′ capping, splicing, editing/modification, and 3′ end processing, are involved in maturation of RNA polymerase II (pol II) transcripts. It is well known that 3′ end cleavage and polyadenylation is essential for most mRNA and long non-coding RNA (lncRNA) expression, stability, localization, and translation. The selection of different polyadenylation sites (PASs) [1–3] leads to production of multiple alternative polyadenylation (APA) isoforms with distinct 3′ untranslated regions (3′ UTRs) and/or protein coding sequences (CDSs). Precise PAS profiling is therefore important to the understanding of gene regulation and function.

Genome-wide PAS profiling was originally performed by examining expressed sequence tags [3,4], and has recently been achieved by specific 3′ end sequencing methods, including but not limited to 3′-seq/3SEQ [5], 3P-seq [6], PAS-seq [7], 3′-READS [8], PolyA-seq [9], and 2P-seq [10]. These 3′ end sequencing methods generate reads enriched in regions close to poly(A) tails owing to the use of oligo(dT) for reverse transcription and 3′ end adapter ligation. In addition, canonical RNA-seq data have also been used for PAS profiling via tools such as DaPars [11,12], as RNA-seq data are also generated from oligo(dT)-enriched poly(A)+ RNAs. With these methods, widespread APA events have been extensively examined in many biological conditions [9,13,14]. For example, cell-type/tissue-specific PAS usage has been suggested to play roles in regulating RNA stability and translation during mouse spermatogenesis [15–17], impacting subcellular transport of RNAs in neurons [18–20], and affecting subcellular location of different protein products translated from mRNA isoforms with distinct 3′ UTRs due to APA [21].

Several PAS databases have been constructed so far for PAS search, analysis, and data download, such as APASdb (http://genome.bucm.edu.cn/utr/) [22], APADB (http://tools.genxpro.net/apadb/) [23], PolyASite (v2.0; https://polyasite.unibas.ch/) [24], PolyA_DB3 (https://exon.apps.wistar.org/polya_db/v3/misc/download.php) [25], and PlantAPAdb (http://www.bmibig.cn/plantAPAdb/) [26]. Moreover, TC3A [12], APAatlas [27], Animal-APAdb [28], and TREND-DB [29] have been constructed by analyzing PASs from poly(A)+ RNA sequencing (RNA-seq) datasets of bulk cells. Despite this progress, most currently available PAS databases are based on bulk cell samples, mainly in human and mouse. Detailed PAS usage at single‐cell resolution and across species has remained largely elusive. Since 3′ tag-based single-cell RNA-seq (scRNA-seq) libraries are prepared from poly(A)+ RNAs captured by oligo(dT), a few bioinformatic methods have been recently developed to detect PASs in single cells, including Sierra [30], scAPA [31], scAPAtrap [32], and SCAPTURE [33], expanding PAS profiling from the bulk‐cell level to the single‐cell level. In addition, two single‐cell PAS databases have been developed: the scAPAatlas database [34] based on the scAPA pipeline and the scAPAdb database [35] based on the Sierra [30] and scAPAtrap [32] pipelines. However, both databases were constructed with a limited number of samples in human, mouse, and plants. It is hence desirable to construct a comprehensive PAS database for multi-species at single‐cell resolution, which will facilitate functional studies of PAS/APA in greater detail.

To achieve this goal, we trained species-specific DeepPASS models, which were further embedded in species-specific SCAPTURE v2 pipelines to profile PASs from 1330 10x Genomics scRNA-seq datasets from a wide spectrum of tissue types in seven species, including human, rhesus macaque, mouse, rat, zebrafish, fruit fly, and worm. With independent nanopore long-read sequencing data from paired human tissues, we validated the vast majority of 3′ UTR PASs, including many previously unannotated ones, confirming the accuracy of PAS calling by SCAPTURE [33]. Taking advantage of this cross-species PAS profiling, we identified PAS usage divergence between orthologous human and mouse genes whose gene expression tends to be cell-type specific. Finally, a comprehensive database of PASs across different species, called PASSpedia, was constructed, which allows users to search, browse, analyze, compare, and download PASs across seven species at single‐cell resolution.

## Data collection and processing

### Data collection of 3′ tag-based scRNA-seq across different species

To annotate single‐cell PASs across different species, we collected 10x Genomics scRNA-seq datasets from Human Cell Atlas (HCA; https://www.humancellatlas.org/), Tabula Sapiens (https://tabula-sapiens.sf.czbiohub.org/), Tabula Muris Senis (https://tabula-muris-senis.ds.czbiohub.org/), Single Cell Expression Atlas (https://www.ebi.ac.uk/gxa/sc/home), FLY CELL ATLAS (https://flycellatlas.org/), and Single Cell Portal (https://singlecell.broadinstitute.org/single_cell), as well as individual publications listed in Table S1 (File S1). In total, 1330 sets of 3′ tag-based scRNA-seq data were obtained, which covered multiple tissues across seven species including human, rhesus macaque, mouse, rat, zebrafish, fruit fly, and worm (Table S1).

**Figure 1:**
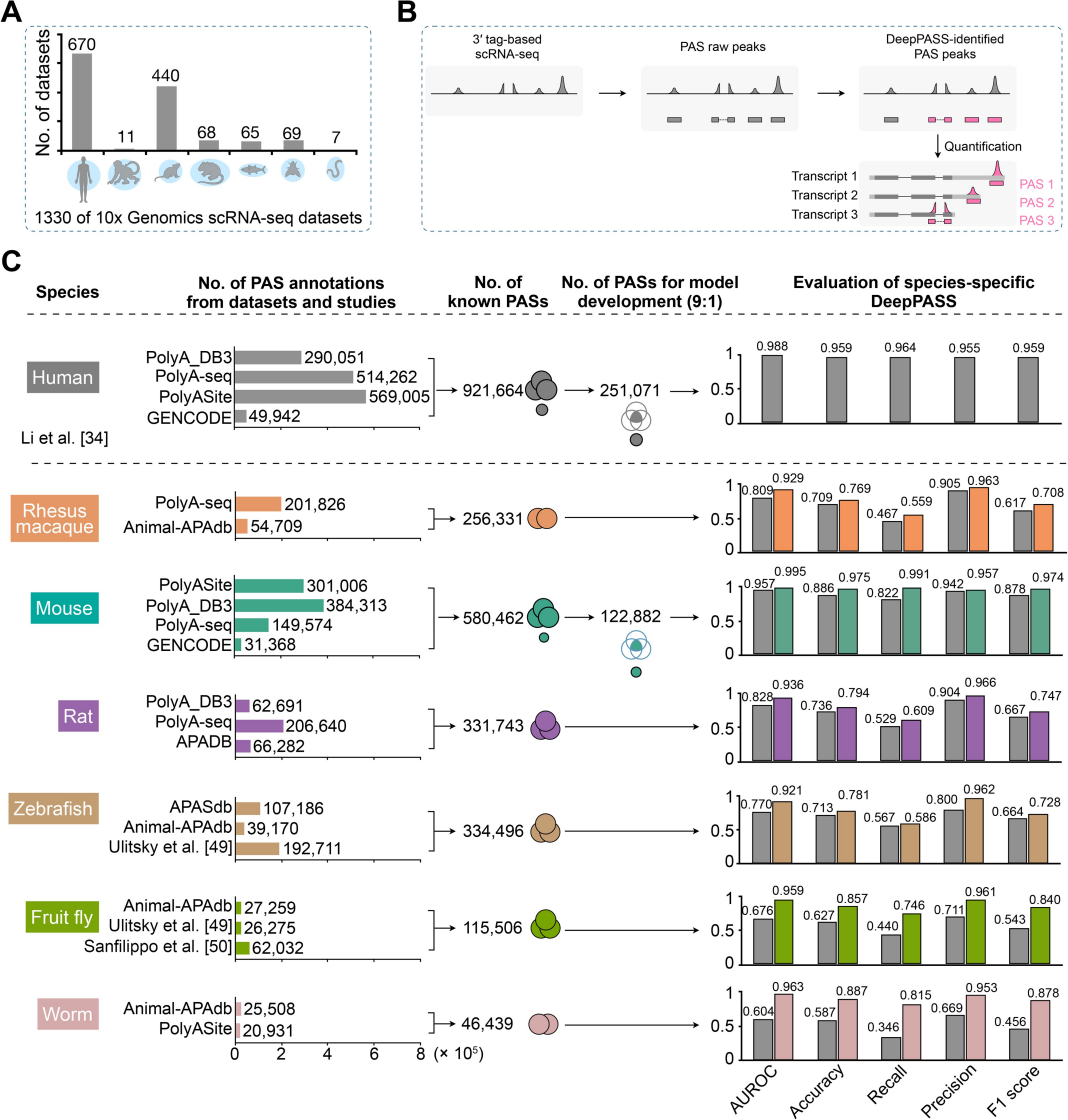
Development of species-specific SCAPTURE v2 pipelines **A**. Collection of 3′ tag-based 10x Genomics scRNA-seq datasets across seven species. **B**. Schematic of the step-wise SCAPTURE pipeline for PAS identification and quantification from scRNA-seq data. **C.** Development of species-specific DeepPASS models for species-specific SCAPTURE v2 pipelines. Collection and selection of known PASs for species-specific DeepPASS model training are shown in left and middle panels, respectively. Evaluation of species-specific DeepPASS models is shown as colored bars in the right panel. Of note, gray bars in the right panel represent original human DeepPASS model performance outcomes for PAS identification in human or other corresponding species. scRNA-seq, single-cell RNA sequencing; PAS, polyadenylation site; AUROC, area under the receiver operating characteristic curve.

Of note, the vast majority of the datasets in this collection were from human and mouse. More specifically, the human collection contained 670 scRNA-seq datasets from 43 studies, amounting to 2,699,298 single cells in total, and the mouse collection contained 440 scRNA-seq datasets from 30 studies, amounting to 1,555,482 single cells in total (Figure S1A). Fewer datasets were obtained from other species, including rhesus macaque, rat, zebrafish, fruit fly, and worm, due to their limited availability (**Figure 1**A, Figure S1A).

### Development of species-specific SCAPTURE pipelines for PAS calling

We previously developed the SCAPTURE pipeline, embedded with a deep learning model DeepPASS, for efficient and precise human PAS calling and quantification from 3′ tag-based scRNA-seq data [33] (Figure 1B). However, direct application of the original DeepPASS model trained with known human PASs for non-human PAS prediction and evaluation in other species, including rhesus macaque, mouse, rat, zebrafish, fruit fly, and worm, exhibited lower performance (gray bars in Figure 1C, right), suggesting sequence variations of PASs across species [28]. Thus, species-specific DeepPASS models are needed for accurate PAS evaluation across different species.

To train species-specific DeepPASS models, we downloaded known PASs from several databases, including Animal-APAdb [28], GENCODE (https://www.gencodegenes.org/), and PolyA_DB3 [25], across different species (bottom panel of Figure 1C; Table S2). Since known PASs were as abundant in mouse as in human, high-confidence mouse PASs were obtained by combining those overlapping in all three databases (PolyASite [24], PolyA_DB3 [25], and PolyA-seq [9]) with those in GENCODE, while PASs in the other non-human species were directly combined from all available databases (Figure 1C). As previously reported [33], known PASs in each of these species were split at a 9:1 ratio between a model training set and an independent validation set. In addition, input sequences were extracted through shifting around training PASs with a normal distribution for model construction after the removal of redundant sequences (File S1) [33]. Evaluation of these species-specific DeepPASS models suggested that each of them (rhesusDeepPASS, mouseDeepPASS, ratDeepPASS, zebrafishDeepPASS, fruitflyDeepPASS, or wormDeepPASS) achieved improved performance on PAS calling with their corresponding species-specific DeepPASS models than those with the original human DeepPASS model (comparing color bars with gray bars in Figure 1C). For example, species-specific SCAPTURE demonstrated improved classification abilities, with increased area under the receiver operating characteristic curve (AUROC) from 0.809 to 0.929, 0.957 to 0.995, 0.828 to 0.936, 0.770 to 0.921, 0.676 to 0.959, and 0.604 to 0.963, respectively, in rhesus macaque, mouse, rat, zebrafish, fruit fly, and worm (Figure 1C).

With corresponding species-specific DeepPASS models, seven species-specific SCAPTURE v2 pipelines were then individually developed and further applied for subsequent PAS calling from 3′ tag-based scRNA-seq datasets (Figure S1B). Of note, exact PAS peaks may exhibit slight differences due to peak-calling biases across multiple scRNA-seq datasets (in different studies and samples). Therefore, PAS integration is essential in multi-sample analyses. As shown in Figure S1C, PAS peaks with at least 60% overlap were considered to originate from the same PAS, and the peak with the highest DeepPASS score among them was selected as the representative PAS for subsequent analyses (Figure S1C; File S1).

**Figure 2:**
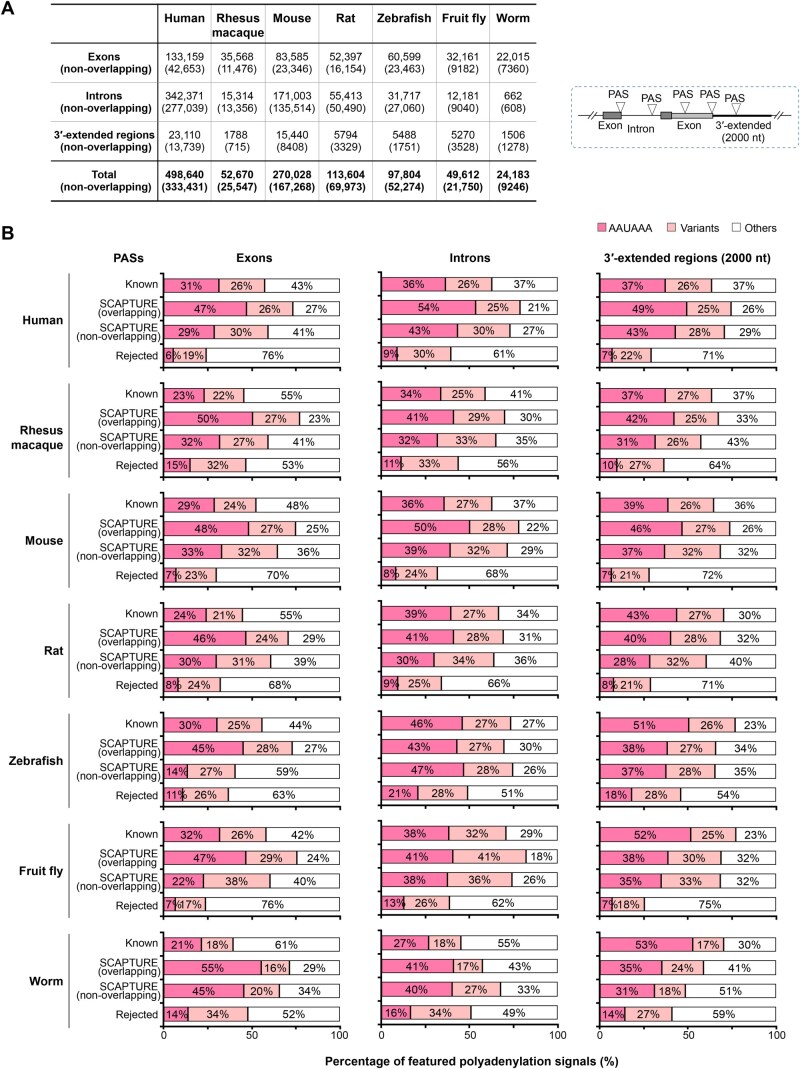
Identification of PASs by species-specific SCAPTURE v2 pipelines at single-cell resolution **A**. Statistics of SCAPTURE-identified PASs from seven species. Left: numbers of exonic, intronic and 3′-extended (within downstream 2000 nt) PASs identified by SCAPTURE v2 across seven species. Right: diagram of exonic, intronic, and 3′-extended PAS classification. Of note, 3′-extended PASs were located within downstream 2000-nt regions of annotated genes. Numbers in parentheses indicate SCAPTURE-identified PASs that do not overlap with known PASs. **B**. Percentages of the canonical AAUAAA motif and its variants in known, SCAPTURE-identified, and rejected PASs in different regions across seven species. The AAUAAA variant motifs include AUUAAA, A[G/C]UAAA, and AA[G/C]AAA. All SCAPTURE-identified PASs were divided into two subgroups: ones overlapping with known PASs and the others non-overlapping with known PASs. The rejected PASs are raw PASs with DeepPASS scores less than 0.5, which were rejected by the DeepPASS model embedded in SCAPTURE.

In total, 498,640 (in human) to 24,183 (in worm) of SCAPTURE-identified PASs were obtained across seven species, each individually located in annotated exonic (Table S3), intronic (Table S4), or intergenic (here 3′-extended 2000 nt only, Table S5) regions (**Figure 2**A). As previously reported [33], typical polyadenylation motifs could be profiled from the context sequence of PASs identified by species-specific SCAPTURE pipelines. These included featured polyadenylation signals, AAUAAA and its variants (AUUAAA, A[G/C]UAAA, and AA[G/C]AAA) located upstream of the cleavage site, as well as downstream U-rich motifs (Figure 2B, Figure S2). Of note, the proportion of featured polyadenylation signals in the upstream regions of SCAPTURE-identified PASs was comparable to or even higher than those of known PASs in all three categories (Figure 2B), supporting the reliability of species-specific SCAPTURE pipelines for PAS calling across species.

### Characterization and validation of SCAPTURE-identified exonic PASs

**Figure 3:**
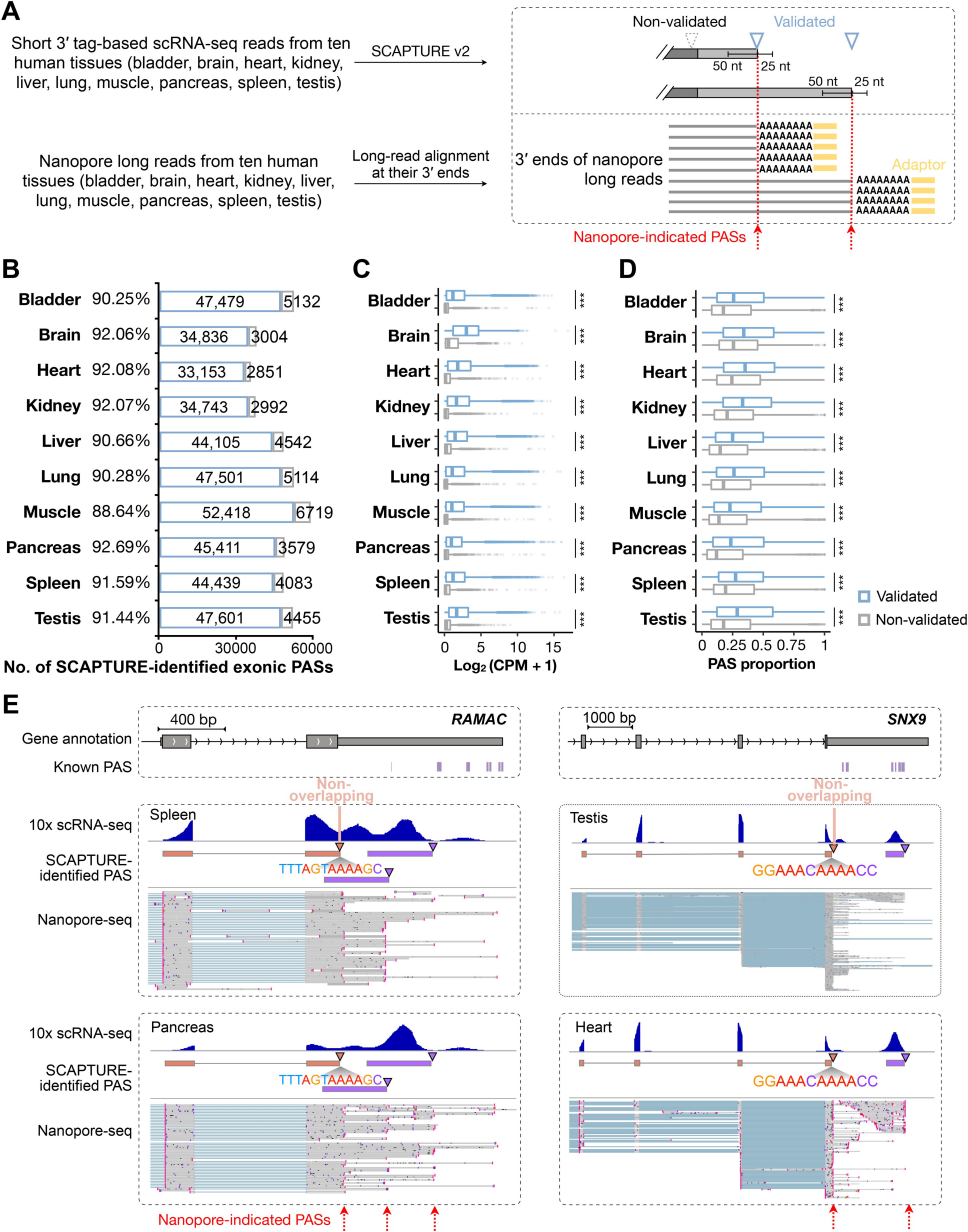
Validation of SCAPTURE-identified exonic PASs with nanopore long-read sequencing data **A**. Schematic of exonic PASs identified by SCAPTURE v2 from short-read scRNA-seq data or by aligning nanopore long-read sequencing data. **B**. Statistics of SCAPTURE-identified exonic PASs validated or non-validated by nanopore long-read sequencing data among ten human tissues. The percentages of validated PASs in all human tissues were labeled next to tissue names. **C**. Expression levels of validated or non-validated exonic PASs among ten human tissues. **D**. Usage of validated or non-validated exonic PASs among ten human tissues. PAS proportion was calculated to represent the usage level of a given PAS, by dividing the expression level of the given PAS by the total expression levels of all identified PASs at the same gene locus. **E**. Examples of validated PASs by nanopore long-read sequencing data. Gene annotation, known PAS annotation, wiggle tracks of 3′ tag-based scRNA-seq data, SCAPTURE-identified PAS peaks, and alignments of nanopore long-read sequencing data at the *RAMAC* and *SNX9* loci in different human tissues are shown for validation. Three PASs, including a non-overlapping (orange, with the AGUAAA motif) and two overlapping (purple) ones, were identified by SCAPTURE v2 from 3′ tag-based scRNA-seq data in the human *RAMAC* locus. Two PASs, including a non-overlapping (orange, with the AACAAA motif) and an overlapping (purple) one, were identified by SCAPTURE v2 from 3′ tag-based scRNA-seq data in the human *SNX9* locus. All these five SCAPTURE-identified PASs were validated by nanopore long-read sequencing data (red arrows). Statistical significance was assessed with Student’s *t*-test (***, *P* < 0.001). CPM, counts per million.

As for SCAPTURE-identified exonic PASs (Table S3), the majority (∼ 60%–70%) of them were found to overlap with known PASs, while about 30%–40% were non-overlapping, across different species (Figure S3A). Next, we set out to further validate SCAPTURE-identified exonic PASs by taking advantage of independent nanopore long-read sequencing datasets. In theory, nanopore long reads with both oligo(A) and adaptor sequences at their 3′ ends are selected for pileup, and their 3′ terminal alignment sites can be used to indicate PASs (**Figure 3**A; File S1). If a SCAPTURE-identified PAS could overlap with a nanopore-indicated PAS within its upstream 50-nt to downstream 25-nt region, this SCAPTURE-identified PAS was then classified as a validated one (Figure 3A). Of note, only a subset of SCAPTURE-identified human exonic PASs, which were detected from 3′ tag-based scRNA-seq of ten human tissue samples with corresponding nanopore long-read sequencing data [36], were selected for this validation. As shown in Figure 3B, about 90% of SCAPTURE-identified human exonic PASs could be validated by long-read sequencing in each comparison of paired human tissue samples, indicating the reliability of using SCAPTURE for PAS calling [33].

Further comparison between validated and non-validated PASs showed that the expression levels [quantified by counts per million (CPM)] [33] of validated PASs were significantly higher than those of non-validated ones in all ten examined human tissues (Figure 3C). In addition, validated PASs were more frequently used than non-validated ones (Figure 3D), evaluated by PAS proportion, which calculates the percentage of a given PAS expression divided by total expression of all PASs at the same gene locus (File S1) [37]. These results thus suggested that the successful detection of PASs is affected by their expression levels and proportions of usage. In line with this notion, since the general expression level of overlapping PASs identified by SCAPTURE from scRNA-seq was significantly higher than that of non-overlapping ones (Figure S3B), over 95% of overlapping PASs could be validated with long-read data, compared to about 55%–60% of non-overlapping ones (Figure S3C). As expected, when filtered by expression levels with CPM ≥ 5, about 75%–85% of highly expressed non-overlapping PASs could be validated with long-read data (Figure S3D), further confirming that successful profiling and validating of PASs are dependent on their expression and usage.

Nevertheless, some validated PASs, including both overlapping (highlighted in purple) and non-overlapping (highlighted in orange) ones, at two genomic loci (*RAMAC* and *SNX9*) are shown in Figure 3E. Since both the overlapping and non-overlapping PASs at the *RAMAC* and *SNX9* loci exhibited different levels of selection/expression among examined tissues, the potential biological regulation by different PAS selections at these gene loci requires further investigation.

### Analyses of SCAPTURE-identified PASs in intronic and 3′-extended regions

Other than high-confidence exonic PASs, a large number of intronic PASs (Table S4) were also identified at single-cell resolution, especially in human and mouse samples (Figure 2A). Although featured polyadenylation signal motifs were revealed in the context sequences of SCAPTURE-identified intronic PASs (Figure 2B), the difficulties in intronic PAS calling due to internal priming of oligo(dT) at A-rich sequences may lead to false-positive PAS detection in intronic regions [30,38]. Consistently, a significant number of poly(A) tracts could be identified within the flanking 300-nt region surrounding the SCAPTURE-identified intronic PASs, much higher than those flanking SCAPTURE-identified exonic PASs (Figure S4A). Of note, it has also been suggested that approximately 50% of 10x Genomics scRNA-seq reads come from intronic regions, due to the binding of intronic poly(A) (8 nt or longer) tracts with oligo(dT) priming during 10x Genomics scRNA-seq library preparation (https://www.10xgenomics.com/cn/support/single-cell-gene-expression/documentation/steps/sequencing/interpreting-intronic-and-antisense-reads-in-10-x-genomics-single-cell-gene-expression-data). Thus, caution is needed for further characterization of these intronic PASs.

In addition, quantified PAS values from 3′ tag-based scRNA-seq further suggested lower expression levels of intronic PASs than those of exonic ones (Figure S4B), while the degrees of tissue specificity of intronic PASs, evaluated by the *Tau* index (File S1), were higher than those of exonic PASs (Figure S4C) in nearly all examined tissues. As the successful detection of exonic PASs was affected by their expression and usage prevalence among different tissues (Figures 3C and D), we suspected that the generally low expression levels of intronic PASs could also affect their identification and validation.

Further, a small number of SCAPTURE-identified PASs were found to be located in 3′-extended 2000-nt regions with high proportions of featured polyadenylation signal motifs (Figure 2A and B). However, highly enriched poly(A) tracts were observed in these PASs (Figure S5A), along with similar low expression levels (Figure S5B) and high degrees of tissue specificity (Figure S5C). Thus, although the embedded DeepPASS model in SCAPTURE has proven to be robust in addressing the internal priming issue to identify previously unannotated intronic PASs [33], additional experimental evidence is needed to confirm the PASs in introns and 3′-extended regions.

### Conservation analysis of PASs across species

**Figure 4:**
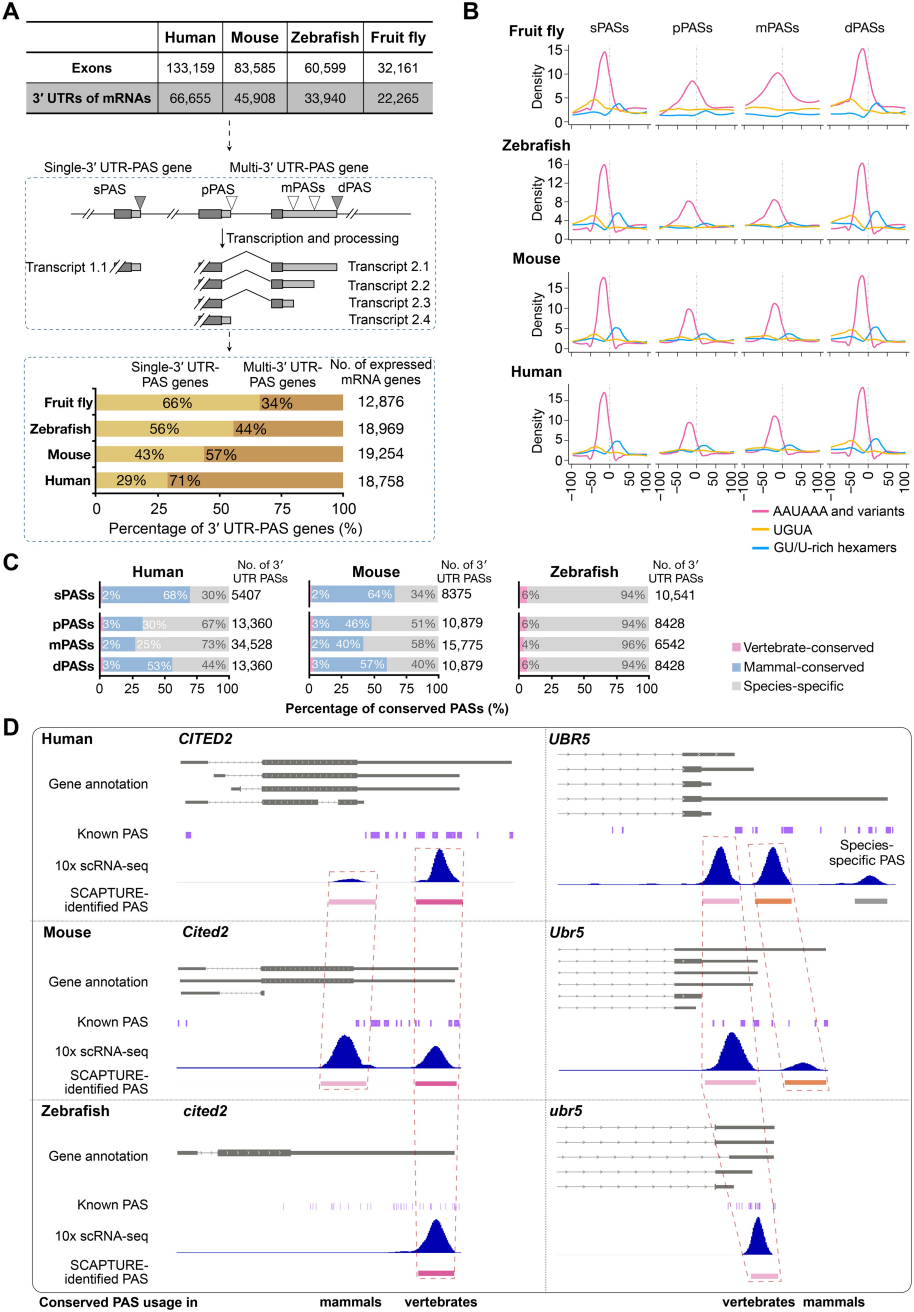
Conservation analysis of PASs **A**. Statistics and classification of 3′ UTR PASs across human, mouse, zebrafish, and fruit fly. Top: selection of SCAPTURE-identified PASs in 3′ UTRs of mRNAs. Middle: classification of single 3′ UTR PASs (sPASs) in single-3′ UTR-PAS mRNA genes or proximal 3′ UTR PASs (pPASs), middle 3′ UTR PASs (mPASs), and distal 3′ UTR PASs (dPASs) in multi-3′ UTR-PAS mRNA genes. Bottom, percentages of single-3′ UTR-PAS and multi-3′ UTR-PAS mRNA genes across human, mouse, zebrafish, and fruit fly. **B**. Distribution of polyadenylation motifs among different 3′ UTR PASs. Canonical AAUAAA motif and its variants (pink), GU/U-rich hexamers (blue), and UGUA (yellow) are shown in identified sPASs, pPASs, mPASs, and dPASs within 200-nt (±100-nt) regions. **C**. Percentages of conserved PASs among human, mouse, and zebrafish. Vertebrate-conserved PASs were identified in all three species of human, mouse, and zebrafish, shown in pink. Mammal-conserved PASs were identified in both human and mouse, but not in zebrafish, shown in light blue. Species-specific PASs were identified only in human, mouse, or zebrafish, shown in gray. **D**. Examples of vertebrate-conserved, mammal-conserved, and human-specific PASs. Left: a mammal-conserved pPAS and a vertebrate-conserved dPAS are indicated in the human *CITED2* locus. Right: a vertebrate-conserved pPAS, a mammal-conserved mPAS, and a human-specific dPAS are individually identified in the human *UBR5* locus. 3′ UTR, 3′ untranslated region.

By taking advantage of single-cell PAS profiling from various tissues and species in this study, we then compared their possible distinct selective usages between different cell types or species. Here, a specific subset of exonic PASs identified in the 3′ UTRs of annotated mRNA genes, called 3′ UTR PASs hereafter, were collected across four model species (human, mouse, zebrafish, and fruit fly) for comparison. Notably, about half to two thirds of exonic PASs were identified in the 3′ UTRs of annotated mRNA genes in these four species (**Figure 4**A, top), while the rest were found predominantly in annotated non-coding RNAs, together with a small number in CDSs or 5′ UTRs of annotated mRNAs (Table S3).

Annotated mRNA genes were classified into two main subgroups based on the number of 3′ UTR PASs: single-3′ UTR-PAS genes, which contain a single 3′ UTR PAS (sPAS) (Figure 4A, middle), and multi-3′ UTR-PAS genes, which contain multiple 3′ UTR PASs, resulting in the expression of multiple APA isoforms with different lengths of 3′ UTRs (Figure 4A, middle). In multi-3′ UTR-PAS genes, PASs closest to the transcription start sites (TSSs) were referred to as proximal 3′ UTR PASs (pPASs for simplicity), those farthest from TSSs as distal 3′ UTR PASs (dPASs for simplicity), and those between pPASs and dPASs as middle 3′ UTR PASs (mPASs for simplicity). Interestingly, the ratio of multi-3′ UTR-PAS genes increased from fruit fly, zebrafish, mouse to human, along with a decreased ratio of single-3′ UTR-PAS genes (Figure 4A, bottom), indicating the additional layer of gene expression regulation provided by alternative selection and usage of multiple 3′ UTR PASs along evolution.

Context sequence analyses showed that distributions of upstream polyadenylation signals, upstream UGUA motifs, and downstream GU/U-rich hexamers were more enriched in sPASs and dPASs than in pPASs and mPASs (Figure 4B), indicating the possible early emergence of sPASs and dPASs before the divergence of mouse and primates. In addition, sPASs and dPASs were shown to be more conserved in sequences between mouse and human than pPASs and mPASs after LiftOver analyses (Figure 4C). However, all these PASs seemed to be much less conserved between zebrafish and human/mouse (Figure 4C), suggesting the rapid evolution of PAS context sequences from non-mammals to mammals. Different patterns and usages of multiple 3′ UTR PASs in human, mouse, and zebrafish brain samples at two gene loci are shown in Figure 4D.

### Distinct PAS usages between human and mouse

We previously showed that quantified PAS values could be used to achieve a similar cell type classification as that achieved by gene expression levels in human scRNA-seq data [33]. Here, with well-known marker genes (File S1), about 80 different cell types were identified from 11 human tissues (including bladder, brain, heart, kidney, intestine, muscle, liver, lung, pancreas, spleen, and testis) as published research [39–41]. In addition, these 80 different cell types were further classified into 9 major subgroups (including 10 types of endothelial cells, 11 types of stromal cells, 10 types of epithelial cells, 4 types of myocytes, 12 types of neuron cells, 2 types of glial cells, 23 types of immune cells, 1 type of hepatocytes, and 7 types of germ cells) (Figure S6A; Table S6).

With the human-specific SCAPTURE, 43,025 3′ UTR PASs could be identified from these 11 human tissues, located in 10,286 multi-3′ UTR-PAS genes (Figure S6B). With the selection criterion of CPM ≥ 5 in more than half of the classified cell types, 5942 highly expressed multi-3′ UTR-PAS genes were selected for cell type clustering analysis (Figure S6B). As previously reported on the use of PASs for successful single-cell classification [33], similar cell classification patterns were achieved by the Uniform Manifold Approximation and Projection (UMAP) dimensionality reduction of the gene expression matrix or by that of corresponding relative dPAS proportions in humans (Figure S6C). Comparable cell type classification was achieved by using the UMAP dimensionality reduction of the expression matrix of 5119 highly expressed multi-3′ UTR-PAS genes or by that of 5119 corresponding mouse dPAS proportions in 11 matched mouse tissues (Figure S7).

**Figure 5:**
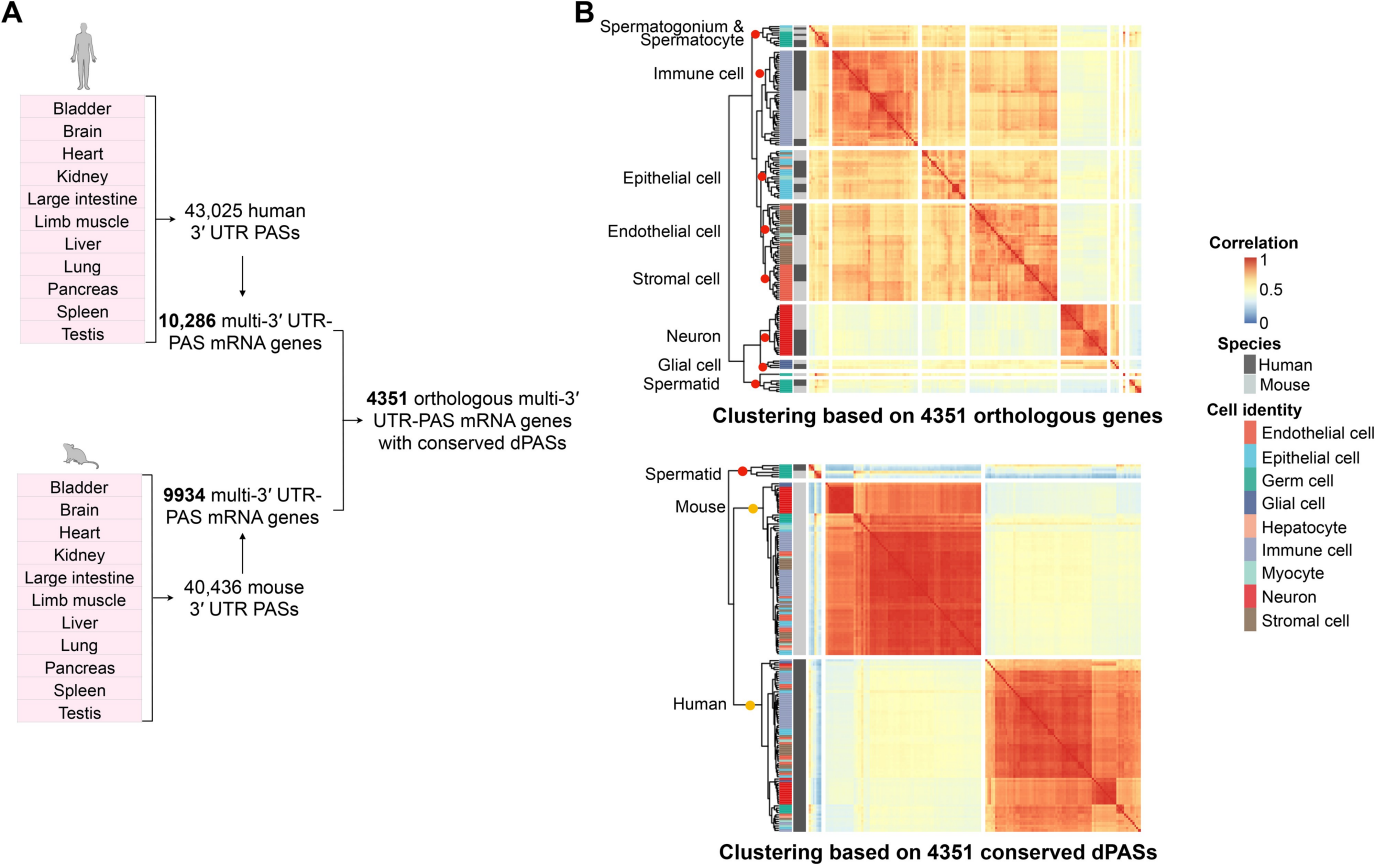
Species-specific PAS usage across human and mouse **A**. Selection of 4351 human-mouse orthologous genes with conserved dPASs. **B**. Different cluster patterns of orthologous gene expression and dPAS usage between human and mouse. Different from the observed intercellular conservation of orthologous gene expression between human and mouse (top), the dPAS usage indicates intraspecies conservation in human or mouse (bottom). The dPAS usage, calculated by dPAS proportion (File S1), in 4351 human-mouse orthologous genes was used for dPAS cluster analysis.

Given the relatively conserved 3′ UTR PAS sequences between human and mouse (Figure 4C), we then set out to further compare their usage levels in matched human and mouse cell types. Between 10,286 human and 9934 mouse multi-3′ UTR-PAS mRNA genes (Table S7) identified in 11 paired human and mouse tissues, 4351 human-mouse orthologous genes with conserved 3′ UTR dPASs were selected for subsequent comparison (**Figure 5**A; Table S8; File S1). Clustering of the 4351 human-mouse orthologous gene expression levels demonstrated intercellular conservation across human and mouse (Figure 5B, top). For example, human immune cells were clustered with mouse immune cells, but not with other types of human cells, such as epithelial and endothelial cells.

In contrast, when using the 4351 corresponding dPASs for clustering, we found that the dPAS proportion has a higher degree of similarity within the same species than intercellular conservation between human and mouse, except for spermatid cells (including round spermatids, elongated spermatids, and sperm) (Figure 5B, bottom), possibly due to the divergent gene expression patterns in testis, with a large fraction of testis-specific gene expression, paralogs, and isoforms [42–44]. The distinct cluster of dPAS proportion from that of gene expression between human and mouse suggests species-specific regulation of dPAS selection, providing an additional layer of gene expression regulation that is worth further investigation.

## Database content and usage

**Figure 6:**
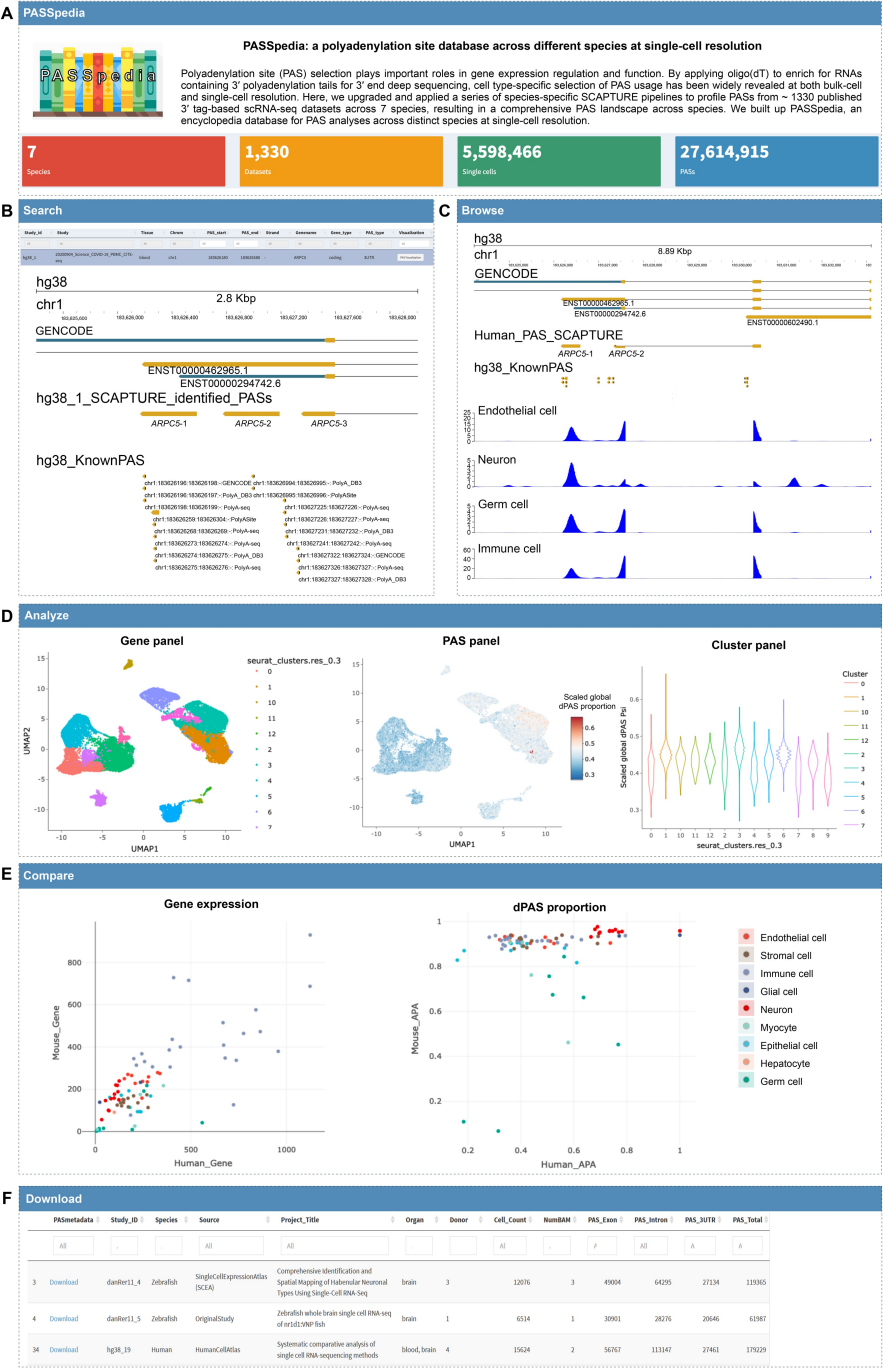
Construction of PASSpedia for cross-species PAS annotation at single-cell resolution **A**. Basic statistics of the PASSpedia database. **B**. The “Search” module in PASSpedia. After searching for PASs across different tissues or experimental groups, a table ﬁle including study_id, study, tissue, chromosome, PAS location, strand, gene name, gene type, and PAS type is generated. In addition, it also provides a “PAS Visualization” link to enable users to visualize SCAPTURE-identified PASs in the genome browser, together with gene annotation and known PASs. **C**. The “Browse” module in PASSpedia. SCAPTURE-identified PASs can be browsed in different cell types together with their wiggle tracks. For example, in the human *ARPC5* gene locus [51–53], a series of *ARPC5* RNA transcripts with different PAS usages can be identified in selected tissues and cell types. Of note, a predominant dPAS usage in neuron cells and a predominant pPAS usage in immune cells were observed in the human *ARPC5* locus. **D**. The “Analyze” module in PASSpedia. Cell clusters of a gene panel by UMAP plot (left), cell clusters of a PAS panel by scaled dPAS proportion (middle), and the global dPAS proportion in each cell cluster by violin plot (right) can be generated after analyzing scRNA-seq datasets from different tissues in a single species. **E**. The “Compare” module in PASSpedia. Scatterplots of gene expression and dPAS usage can be generated after comparing scRNA-seq datasets from two different species, exemplified here by comparing *ARPC5* gene expression (left) and dPAS proportion (right) between human and mouse. Each dot represents a selected cell type. **F**. The “Download” module in PASSpedia. SCAPTURE-identified PASs can be further downloaded from selected studies or an integrated set of PASs in each species for further investigation. *ARPC5*, actin related protein 2/3 complex subunit 5; UMAP, Uniform Manifold Approximation and Projection; APA, alternative polyadenylation.

To facilitate PAS-related studies, we further developed a user-friendly database, PASSpedia (**Figure 6**A), which includes all PASs identified by SCAPTURE from over 5 million single cells across 7 species, derived from 1330 datasets of 99 studies. PASSpedia consists of five modules, including PAS searching, browsing, analyzing, comparing, and downloading.

First, PASSpedia offers a basic “Search” module (Figure 6B) across different tissues and/or studies. This module provides a table including study information and PAS location, as well as a “PAS Visualization” button to visualize the PAS, gene annotation, and known PASs in a genome browser.

Second, PASSpedia provides a “Browse” module, by which users can select a gene and cell type of interest, and then click the “Visualization” button to display the wiggle track of all PASs for the given gene locus in selected cell types within the same species (Figure 6C).

Third, PASSpedia also contains an “Analyze” module to show both gene expression matrix and PAS proportion among different cell types from individual studies (Figure 6D). With the inclusion of massive scRNA-seq datasets from different tissues and species in PASSpedia, the UMAP plots of single-cell clustering by gene expression and the scaled dPAS (or pPAS) proportion can be plotted at single-cell resolution (Figure 6D, left and middle). Overall PAS (dPAS or pPAS) preference across different cell clusters in each scRNA-seq sample can be visualized (Figure 6D, right).

Fourth, PASSpedia features a “Compare” module to show both gene expression and PAS usage differences of one selected gene (such as *ARPC5*) between two species (Figure 6E). Both gene expression (Figure 6E, left) and dPAS usage (Figure 6E, right) levels of the selected gene can be visualized from different cell types of selected tissues and cell classes between two species.

Finally, PASSpedia also contains a “Download” module, where users can download PASs from selected studies or integrated sets of PASs in each species for customer-preferred analyses (Figure 6F).

## Discussion

In this study, we expanded our previously developed SCAPTURE pipeline (v1 to v2) for species-specific PAS identification and evaluation (Figure 1), achieving a comprehensive collection of PASs with featured polyadenylation signals at single-cell resolution across seven species (Figure 2). However, the model performance in non-human/mouse species remains lower than that in human and mouse, largely due to the lack of reliable PAS annotations for model development. In the future, as more reliable PASs become available, better species-specific models could be further developed, especially in those non-human/mouse species. With nanopore long-read sequencing data from ten matched tissues (Figure 3), the vast majority of SCAPTURE-identified exonic PASs in ten tissues could be validated, supporting the reliability and robustness of SCAPTURE for PAS calling among massive tissues or cell types.

Of note, although SCAPTURE has been shown to have high sensitivity and specificity for intronic PAS calling from 3′ tag-based scRNA-seq data [33], the internal priming issue appears to substantially affect intronic PAS calling [30,38]. Also in line with the fact that up to half of all 10x Genomics scRNA-seq reads could come from intronic regions due to the binding of intronic poly(A) tracts to oligo(dT) during reverse transcription, a significant number of SCAPTURE-identified intronic PASs were shown to be associated with poly(A) tracts in their context regions. In addition, intronic PAS calling is affected by the generally low and often tissue-specific expression of these PASs (Figure S4). Therefore, despite high ratios of featured polyadenylation signals in SCAPTURE-identified intronic PASs for known intronic ones (Figure 2), careful examination and caution are needed before further analysis of intronic PASs.

Nevertheless, analyzing massive 3′ tag-based scRNA-seq datasets from a wide spectrum of tissues across different species contributed to the identification of previously unannotated PASs with SCAPTURE (Figures 2 and 3). By taking advantage of this PAS profiling, cross-species comparison showed that more multi-3′ UTR-PAS genes could be identified along evolution, such as from fruit fly and zebrafish to mouse and human (Figure 4). Furthermore, sequence conservation of PASs was shown to be very low between non-mammals (such as zebrafish and worm) and mammals (such as mouse and human). Additional analysis between human and mouse suggested relatively higher conservation of sPASs and dPASs than that of pPASs and mPASs (Figure 4C). Despite sequence conservation, clustering analyses demonstrated that the dPAS usage exhibited intraspecies conservation in human or mouse, while their orthologous gene expression showed conservation in an intercellular manner across human and mouse (Figure 5). This suggests a species-specific 3′ UTR PAS usage and regulation that is independent of gene expression, similar to the observed species-dominated clustering of alternative splicing events in multiple vertebrate lineages [45,46]. Detailed understanding of species-specific regulation of PAS selection and its biological roles awaits further investigation. Interestingly, a recent publication also reported that changes in gene expression and 3′ UTR length (suggesting different PAS usages) were independent among diverse cell types of human and mouse [47]. Of note, another post-/co-transcriptional regulatory mechanism, alternative splicing, has also been shown to respond to species-specific features more than to tissue-specific conservation [45,46]. These findings together underscore that, in addition to gene expression, other regulatory effects, such as alternative splicing and APA, also shape transcriptomic complexity and further expand their functional diversity.

To facilitate PAS and APA studies, we built the PASSpedia database for PAS searching, browsing, analyzing, comparing, and downloading from different tissues and cell types (Figure 6). The PASSpedia database also features customized comparison of distinct PAS usages and their corresponding transcript expression between different cell types, tissues, and species.

In summary, with the widespread adoption of 3′ tag-based scRNA-seq, a detailed PAS atlas, PASSpedia, has been constructed from multiple cell types and tissues across distinct species, providing comprehensive PAS profiling and dynamic PAS usage at single-cell resolution. In the future, the rapid accumulation of 3′ tag-based single-cell and spatial RNA-seq datasets will allow even more detailed and context-specific profiling of PASs at single-cell resolution. Such datasets will not only enhance our understanding of PAS regulatory roles in specific tissues and cell types but also provide the foundation for comparative studies across species. Extending similar analyses to other organisms covering an even broader range of species, will substantially advance our understanding of PAS usage and offer novel insights into the evolutionary conservation and/or divergence of 3′ PAS selection with phylogenetic support.

## Code availability

SCAPTURE v2 pipeline is accessible at https://github.com/YangLab/SCAPTURE-v2. The code has also been submitted to BioCode at the National Genomics Data Center (NGDC), China National Center for Bioinformation (CNCB) (BioCode: BT007965), which is publicly accessible at https://ngdc.cncb.ac.cn/biocode/tools/BT007965.

## Data availability

SCAPTURE-identified PASs are recorded in PASSpedia (https://bits.fudan.edu.cn/PASSpedia/). PASSpedia has been submitted to Database Commons [48] at the NGDC, CNCB, which is publicly accessible at https://ngdc.cncb.ac.cn/databasecommons/database/id/10240.

## CRediT author statement

**Pei-Hong Zhang:** Conceptualization, Methodology, Software, Formal analysis, Visualization, Writing – original draft. **Hua Feng:** Data curation, Formal analysis, Visualization. **Xu-Kai Ma:** Writing – review & editing. **Fang Nan:** Writing – original draft, Writing – review & editing. **Li Yang:** Writing – original draft, Writing – review & editing, Conceptualization, Supervision, Project administration, Funding acquisition. All authors have read and approved the final manuscript.

## Competing interests

The authors declare no competing interests.

## Supplementary Material

qzaf089_Supplementary_Data
