# Supplementary material for: PASSpedia: A Polyadenylation Site Database Across Different Species at Single‐cell Resolution
Source: Genomics Proteomics Bioinformatics. 2025 Sep 23;24(2):qzaf089. doi: 10.1093/gpbjnl/qzaf089 (PMC13309242; doi:10.1093/gpbjnl/qzaf089)

A

Species	No. of study	No. of datasets	No. of cells
Human	43	670	2,699,298
Rhesus	4	11	64,328
Mouse	30	440	1,555,482
Rat	4	68	330,507
Zebrafish	12	65	308,433
Fruit-fly	5	69	550,717
Worm	1	7	89,701
Total	99	1330	5,598,466

B

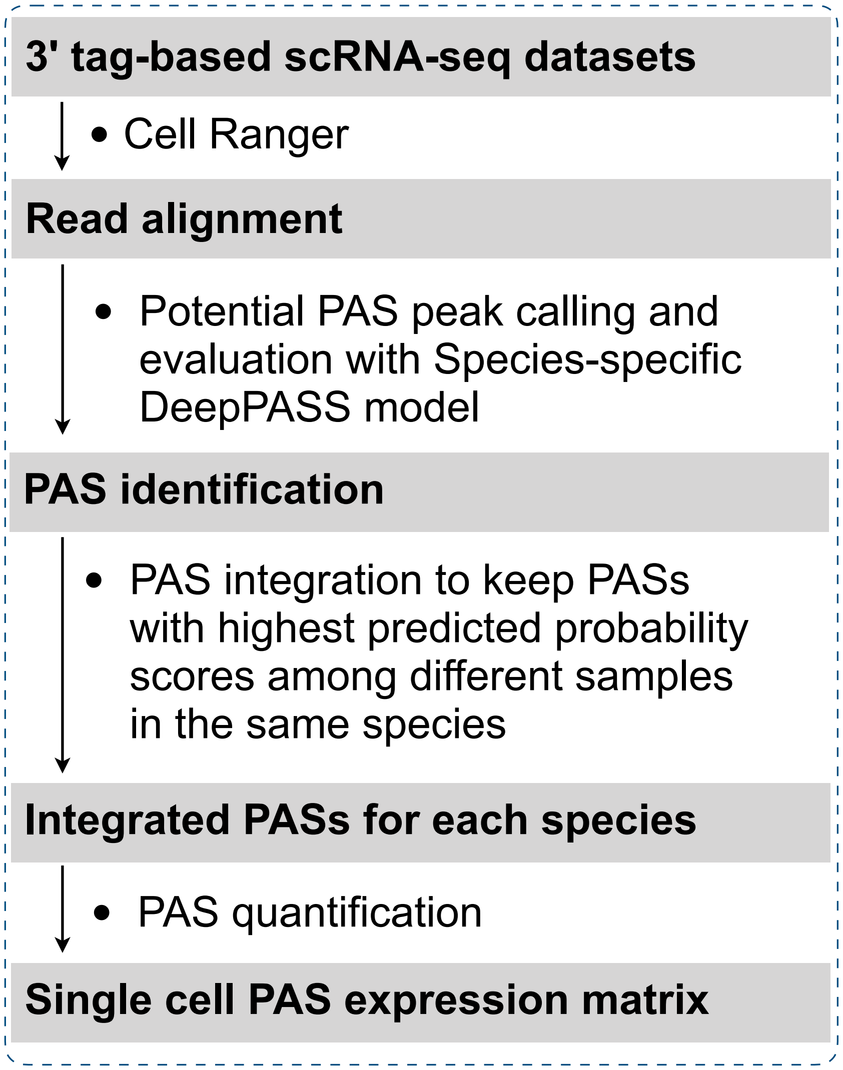

C

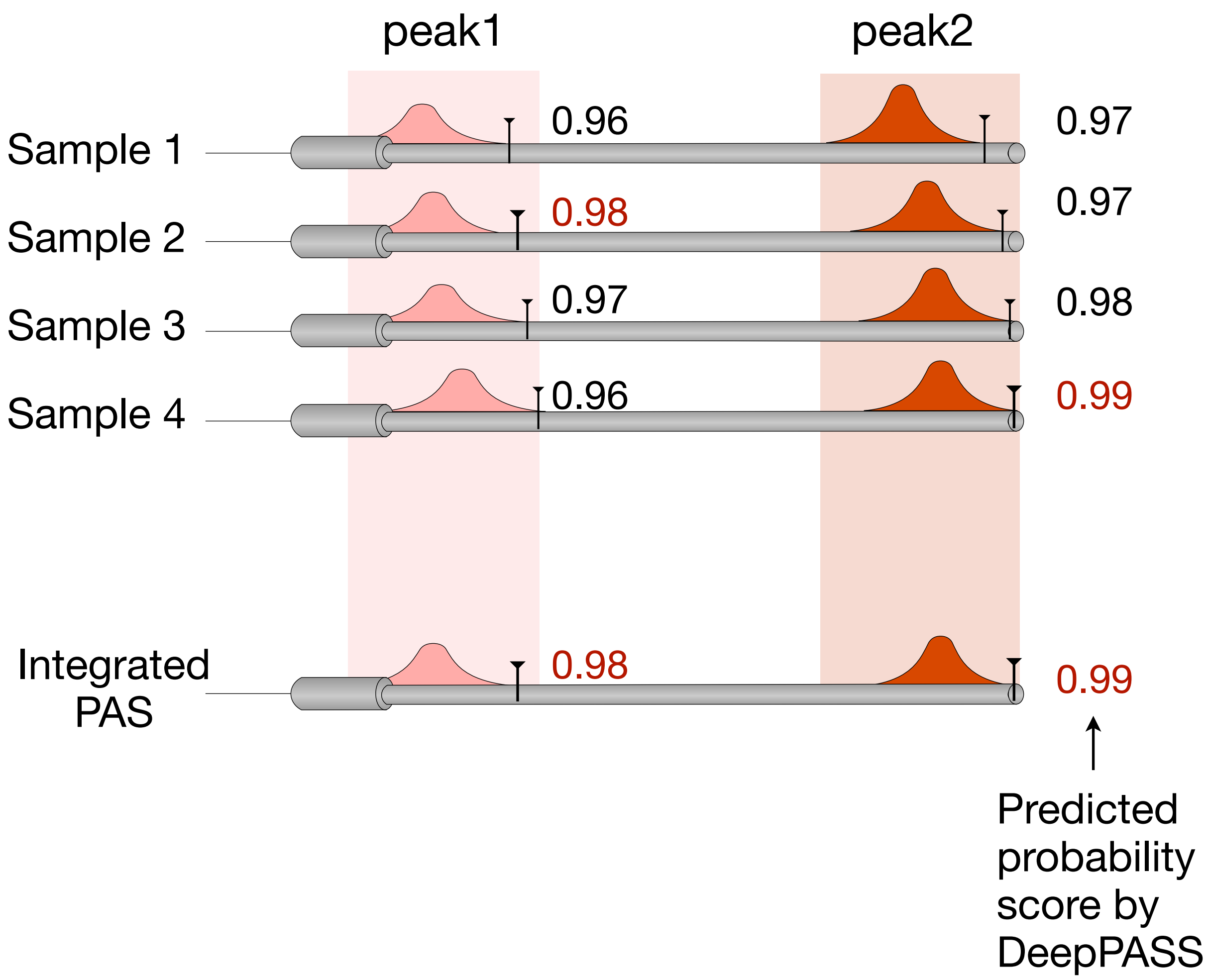

Supplement: qzaf089_Supplementary_Data [file qzaf089_supplementary_data.zip › 14-Apr-2026_091115_figS1_0929.pdf]
