# Supplementary material for: PASSpedia: A Polyadenylation Site Database Across Different Species at Single‐cell Resolution
Source: Genomics Proteomics Bioinformatics. 2025 Sep 23;24(2):qzaf089. doi: 10.1093/gpbjnl/qzaf089 (PMC13309242; doi:10.1093/gpbjnl/qzaf089)
Supplement: qzaf089_Supplementary_Data [file qzaf089_supplementary_data.zip › qzaf089 Supplementary data-new.zip!/FIgS4.pdf]

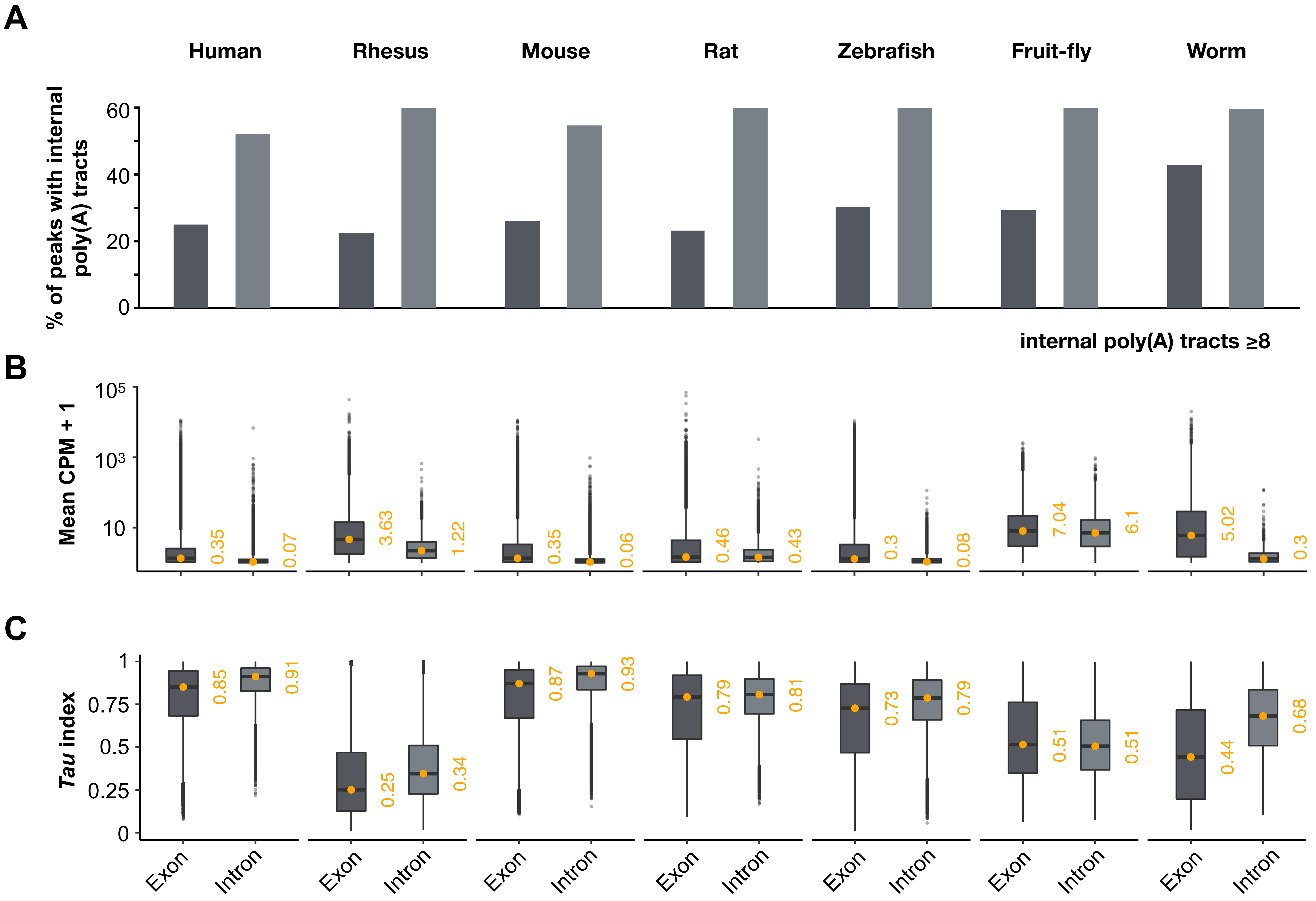

A
Human
Rhesus
Mouse
Rat
Zebrafish
Fruit-fly
Worm
% of peaks with internal poly(A) tracts
0
20
40
60
internal poly(A) tracts ≥8
B
Mean CPM + 1
10
10³
10⁵
0.35
0.07
3.63
1.22
0.35
0.06
0.46
0.43
0.3
0.08
7.04
6.1
5.02
0.3
C
Tau index
0
0.25
0.5
0.75
1
0.85
0.91
0.25
0.34
0.87
0.93
0.79
0.81
0.73
0.79
0.51
0.51
0.44
0.68
Exon
Intron
