# Supplementary material for: PASSpedia: A Polyadenylation Site Database Across Different Species at Single‐cell Resolution
Source: Genomics Proteomics Bioinformatics. 2025 Sep 23;24(2):qzaf089. doi: 10.1093/gpbjnl/qzaf089 (PMC13309242; doi:10.1093/gpbjnl/qzaf089)

A

Species	No. of study	No. of datasets	No. of cells
**Human**	43	670	2,699,298
**Rhesus macaque**	4	11	64,328
**Mouse**	30	440	1,555,482
**Rat**	4	68	330,507
**Zebrafish**	12	65	308,433
**Fruit fly**	5	69	550,717
**Worm**	1	7	89,701
**Total**	99	1330	5,598,466

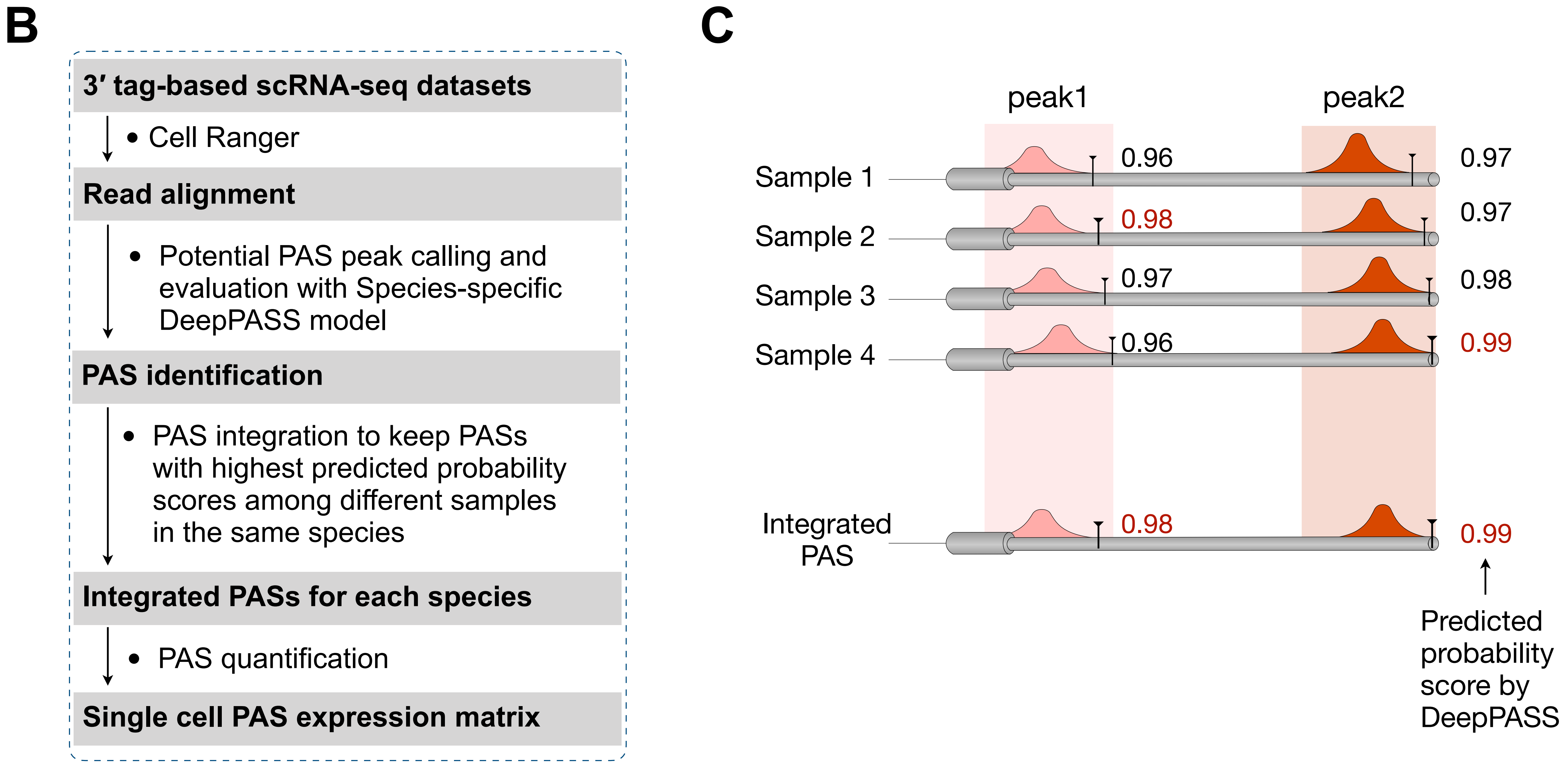

Supplement: qzaf089_Supplementary_Data [file qzaf089_supplementary_data.zip › qzaf089 Supplementary data-new.zip!/FigS1.pdf]
