# Supplementary material for: PASSpedia: A Polyadenylation Site Database Across Different Species at Single‐cell Resolution
Source: Genomics Proteomics Bioinformatics. 2025 Sep 23;24(2):qzaf089. doi: 10.1093/gpbjnl/qzaf089 (PMC13309242; doi:10.1093/gpbjnl/qzaf089)
Supplement: qzaf089_Supplementary_Data [file qzaf089_supplementary_data.zip › qzaf089 Supplementary data-new.zip!/FigS2.pdf]

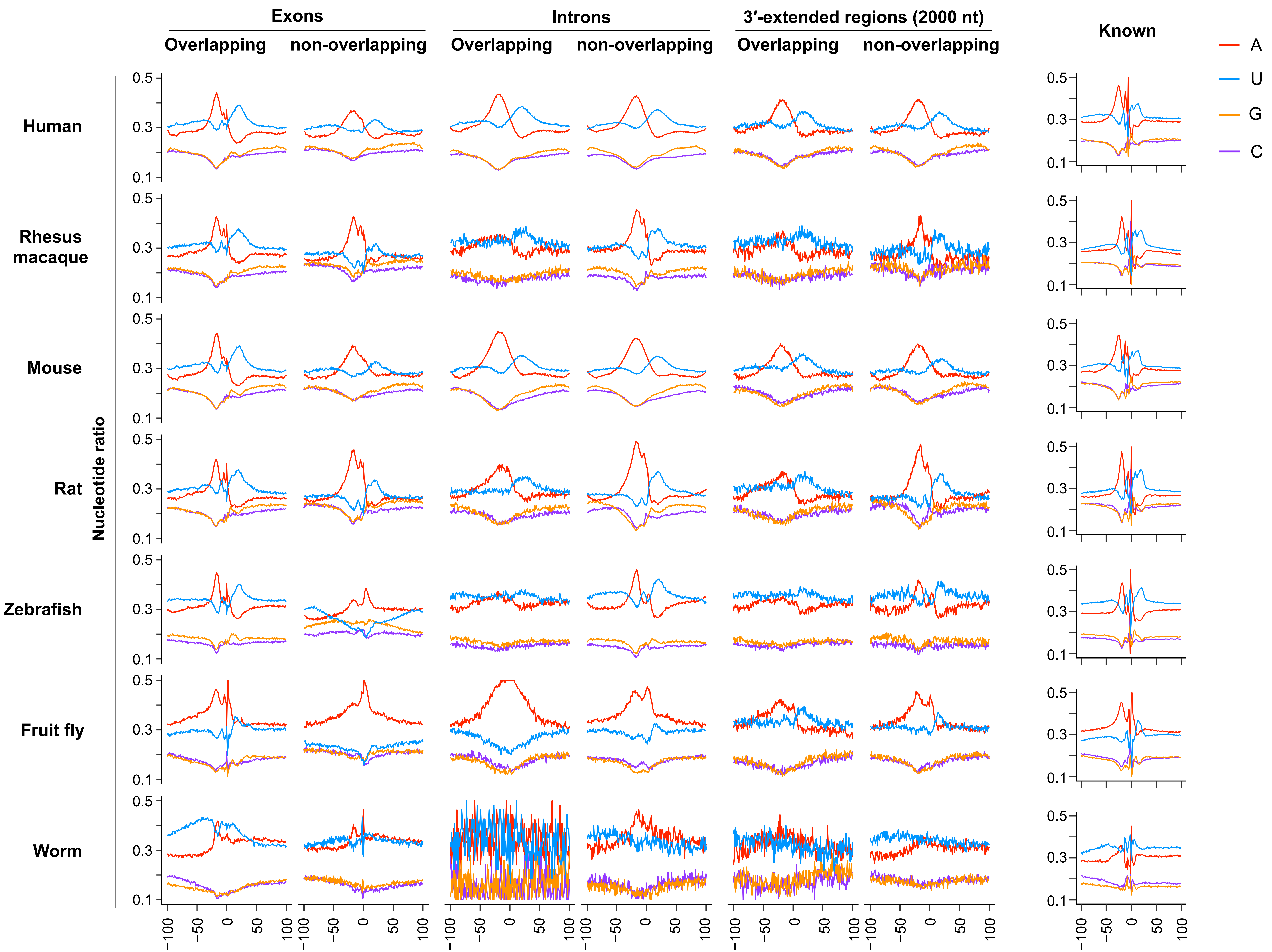
Exons
Introns
3′-extended regions (2000 nt)
Overlapping
non-overlapping
Known
A
U
G
C
Human
Rhesus macaque
Mouse
Rat
Zebrafish
Fruit fly
Worm
Nucleotide ratio
0.5
0.3
0.1
−100
−50
0
50
100
