# Supplementary material for: PASSpedia: A Polyadenylation Site Database Across Different Species at Single‐cell Resolution
Source: Genomics Proteomics Bioinformatics. 2025 Sep 23;24(2):qzaf089. doi: 10.1093/gpbjnl/qzaf089 (PMC13309242; doi:10.1093/gpbjnl/qzaf089)
Supplement: qzaf089_Supplementary_Data [file qzaf089_supplementary_data.zip › qzaf089 Supplementary data-new.zip!/FigS3.pdf]

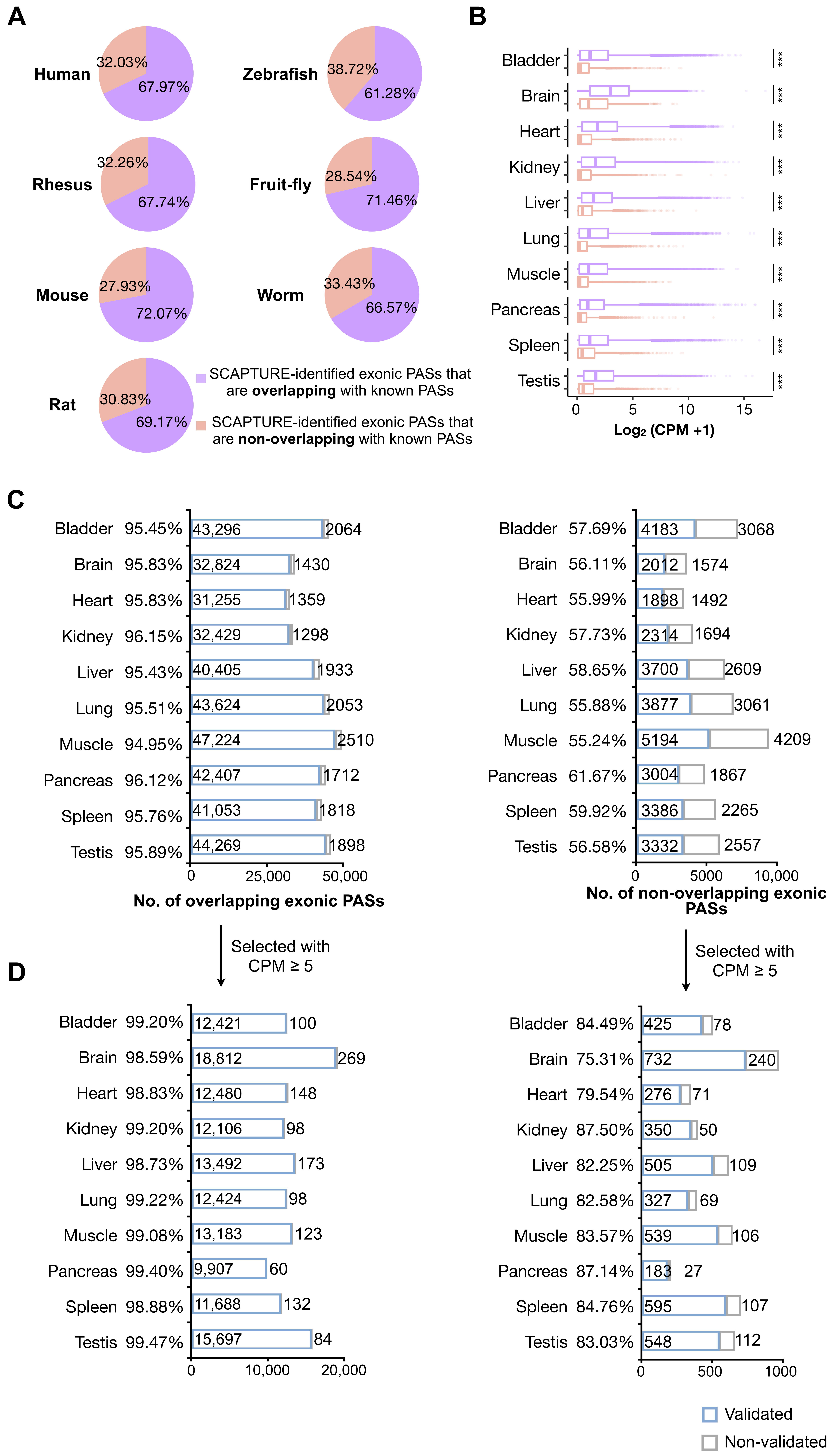

A
Human
32.03%
67.97%
Zebrafish
38.72%
61.28%
Rhesus
32.26%
67.74%
Fruit-fly
28.54%
71.46%
Mouse
27.93%
72.07%
Worm
33.43%
66.57%
Rat
30.83%
69.17%
SCAPTURE-identified exonic PASs that are **overlapping** with known PASs
SCAPTURE-identified exonic PASs that are **non-overlapping** with known PASs
B
Bladder
Brain
Heart
Kidney
Liver
Lung
Muscle
Pancreas
Spleen
Testis
***
0
5
10
15
Log2 (CPM +1)
C
Bladder 95.45% 43,296 2064
Brain 95.83% 32,824 1430
Heart 95.83% 31,255 1359
Kidney 96.15% 32,429 1298
Liver 95.43% 40,405 1933
Lung 95.51% 43,624 2053
Muscle 94.95% 47,224 2510
Pancreas 96.12% 42,407 1712
Spleen 95.76% 41,053 1818
Testis 95.89% 44,269 1898
0
25,000
50,000
No. of overlapping exonic PASs
Bladder 57.69% 4183 3068
Brain 56.11% 2012 1574
Heart 55.99% 1898 1492
Kidney 57.73% 2314 1694
Liver 58.65% 3700 2609
Lung 55.88% 3877 3061
Muscle 55.24% 5194 4209
Pancreas 61.67% 3004 1867
Spleen 59.92% 3386 2265
Testis 56.58% 3332 2557
0
5000
10,000
No. of non-overlapping exonic PASs
Selected with CPM ≥ 5
Selected with CPM ≥ 5
D
Bladder 99.20% 12,421 100
Brain 98.59% 18,812 269
Heart 98.83% 12,480 148
Kidney 99.20% 12,106 98
Liver 98.73% 13,492 173
Lung 99.22% 12,424 98
Muscle 99.08% 13,183 123
Pancreas 99.40% 9,907 60
Spleen 98.88% 11,688 132
Testis 99.47% 15,697 84
0
10,000
20,000
Bladder 84.49% 425 78
Brain 75.31% 732 240
Heart 79.54% 276 71
Kidney 87.50% 350 50
Liver 82.25% 505 109
Lung 82.58% 327 69
Muscle 83.57% 539 106
Pancreas 87.14% 183 27
Spleen 84.76% 595 107
Testis 83.03% 548 112
0
500
1000
Validated
Non-validated
