# Supplementary material for: PASSpedia: A Polyadenylation Site Database Across Different Species at Single‐cell Resolution
Source: Genomics Proteomics Bioinformatics. 2025 Sep 23;24(2):qzaf089. doi: 10.1093/gpbjnl/qzaf089 (PMC13309242; doi:10.1093/gpbjnl/qzaf089)
Supplement: qzaf089_Supplementary_Data [file qzaf089_supplementary_data.zip › qzaf089 Supplementary data-new.zip!/FigS5.pdf]

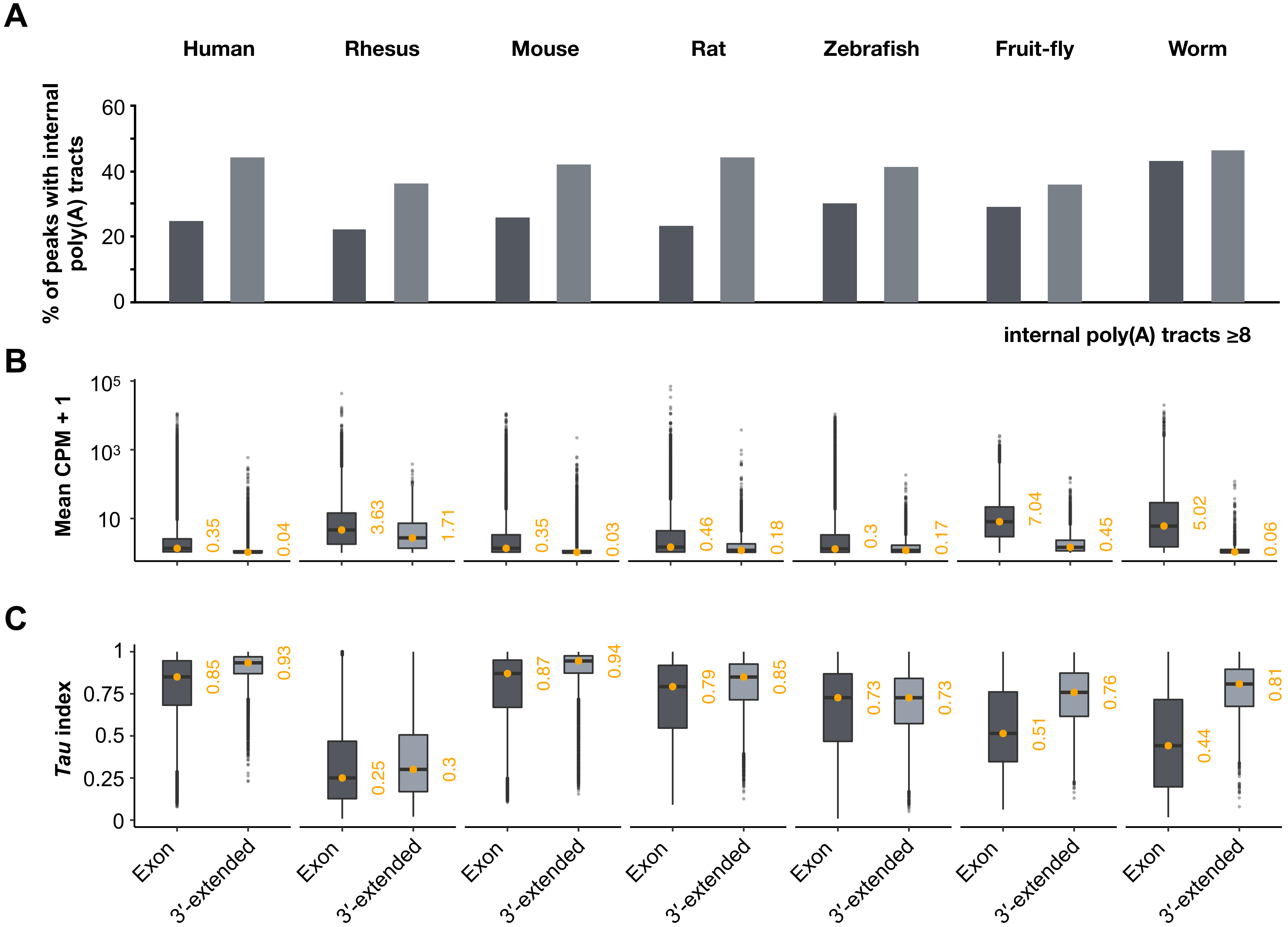
A
Human
Rhesus
Mouse
Rat
Zebrafish
Fruit-fly
Worm
% of peaks with internal poly(A) tracts
60
40
20
0
internal poly(A) tracts ≥8
B
Mean CPM + 1
10⁵
10³
10
0.35
0.04
3.63
1.71
0.35
0.03
0.46
0.18
0.3
0.17
7.04
0.45
5.02
0.06
C
Tau index
1
0.75
0.5
0.25
0
0.85
0.93
0.25
0.3
0.87
0.94
0.79
0.85
0.73
0.73
0.51
0.76
0.44
0.81
Exon
3′-extended
