# Supplementary material for: PASSpedia: A Polyadenylation Site Database Across Different Species at Single‐cell Resolution
Source: Genomics Proteomics Bioinformatics. 2025 Sep 23;24(2):qzaf089. doi: 10.1093/gpbjnl/qzaf089 (PMC13309242; doi:10.1093/gpbjnl/qzaf089)
Supplement: qzaf089_Supplementary_Data [file qzaf089_supplementary_data.zip › qzaf089 Supplementary data-new.zip!/FigS6.pdf]

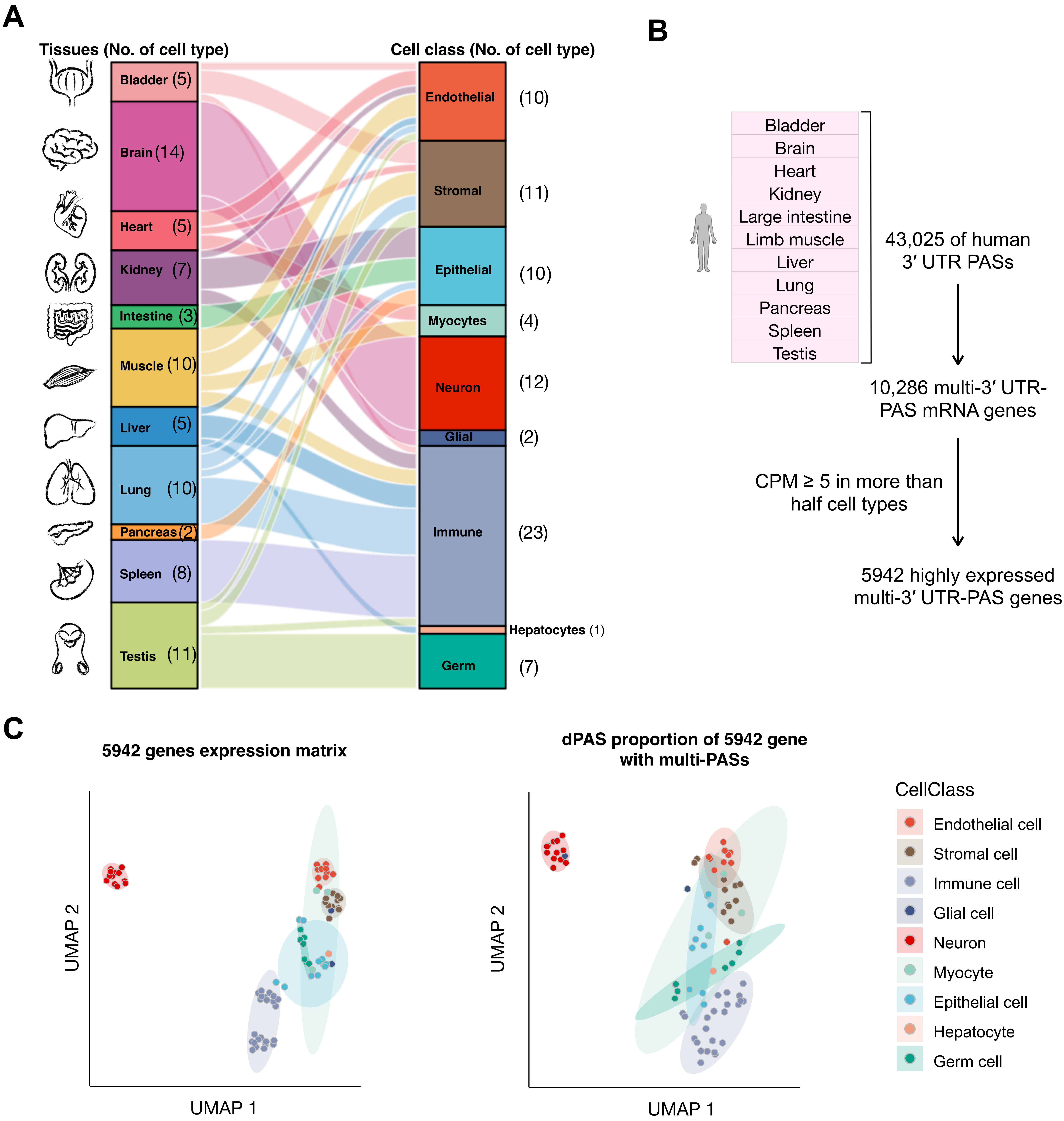
A
Tissues (No. of cell type)
Cell class (No. of cell type)
Bladder (5)
Brain (14)
Heart (5)
Kidney (7)
Intestine (3)
Muscle (10)
Liver (5)
Lung (10)
Pancreas (2)
Spleen (8)
Testis (11)
Endothelial (10)
Stromal (11)
Epithelial (10)
Myocytes (4)
Neuron (12)
Glial (2)
Immune (23)
Hepatocytes (1)
Germ (7)
B
Bladder
Brain
Heart
Kidney
Large intestine
Limb muscle
Liver
Lung
Pancreas
Spleen
Testis
43,025 of human 3′ UTR PASs
10,286 multi-3′ UTR-PAS mRNA genes
CPM ≥ 5 in more than half cell types
5942 highly expressed multi-3′ UTR-PAS genes
C
5942 genes expression matrix
dPAS proportion of 5942 gene with multi-PASs
UMAP 2
UMAP 1
UMAP 2
UMAP 1
CellClass
Endothelial cell
Stromal cell
Immune cell
Glial cell
Neuron
Myocyte
Epithelial cell
Hepatocyte
Germ cell
