# Supplementary material for: PASSpedia: A Polyadenylation Site Database Across Different Species at Single‐cell Resolution
Source: Genomics Proteomics Bioinformatics. 2025 Sep 23;24(2):qzaf089. doi: 10.1093/gpbjnl/qzaf089 (PMC13309242; doi:10.1093/gpbjnl/qzaf089)
Supplement: qzaf089_Supplementary_Data [file qzaf089_supplementary_data.zip › qzaf089 Supplementary data-new.zip!/File S1.docx]

**Supplementary method**

**Collection of scRNA-seq datasets and pre-processing**

All raw FASTQ files or processed BAM files of published 10x Genomics scRNA-seq datasets in totally 99 studies were downloaded from Human Cell Atlas (HCA, https://www.humancellatlas.org/), Gene Expression Omnibus (GEO, https://www.ncbi.nlm.nih.gov/geo), Sequence Read Archive (SRA, https://www.ncbi.nlm.nih.gov/sra), 10x Genomics website (https://www.10xgenomics.com/) or individual publications for a broader tissue coverage, especially in rhesus macaque, rat, and worm (Table S1). Three criteria were applied to select scRNA-seq for our analysis. First, since 3′ tag-based sequencing strategy is essential for the identification of PASs at the single cell level, we only chose 3′ tag-based scRNA-seq data for our study, while many 10x Genomics datasets primarily capture the 5′ ends were excluded. Second, SCAPTURE pipeline requires bam/fastq files to call peaks that were further evaluated by embedded DeepPASS models to identify PASs. Thus, a lot of previously processed datasets without bam/fastq files were excluded. Third, redundant datasets, such as those from blood, were also removed to ensure the balance of sample numbers among deferent tissues. Of note, all these datasets were achieved from single cells, except human neuron datasets that were performed from single nucleus RNA-seq datasets [1].

All scRNA-seq datasets were analyzed by Cell Ranger software (v6.0.1) [2] according to the 10x Genomics official document (<https://www.10xgenomics.com/>). Before alignment, genome reference of each species was built using Cell Ranger *mkref* with genome and paired gene annotation with default parameters. Cell Ranger *count* with default parameters was applied for genome alignment, cell barcode identification and UMIs incorporation. Generated gene-cell barcode matrices were used for downstream scRNA-seq analysis using the R package Seurat (v4.0.3) [3]. BAM files were used to identify PASs with SCAPTURE v2 pipeline (see below).

**Collection of reference genomes and gene annotations for seven species**

Genomic references and gene annotations of seven model animals (Table S2), including Homo sapiens (human, GRCh38/hg38), Macaca mulatta (Rhesus macaque, Mmul8.0/rheMac8), Mus musculus (mouse, GRCm38/mm10), Rattus norvegicus (rat, RGSC6.0/rn6), Danio rerio (zebrafish, GRCz11/danRer11), Drosophila melanogaster (fruit fly, BDGP Release 6+ ISO1 MT/dm6) and Caenorhabditis elegans (worm, WS235/ce11), were derived from databases including GENCODE (https://www.gencodegenes.org/), RefSeq (NCBI Reference Sequence Database, https://www.ncbi.nlm.nih.gov/refseq) and Ensembl (https://www.ensembl.org/). Gene annotations of rhesus macaque, rat, zebrafish, fruit fly, and worm, which obtained from both RefSeq and Ensembl databases, were then deduplicated and merged based on gene names and transcript information. Orthologous genes of human and mouse were obtained from the Ensembl database.

**Collection of known PASs in seven species**

Known PASs in seven species were obtained from publicly available databases, including GENCODE (https://www.gencodegenes.org/), APASdb [4] (http://genome.bucm.edu.cn/utr/), Animal-APAdb [5] (http://gong_lab.hzau.edu.cn/Animal-APAdb/), PolyASite [6] (v2.0, https://polyasite.unibas.ch/), PolyA-seq [7], and PolyA_DB3 [8] (https://exon.apps.wistar.org/polya_db/v3/misc/download.php), and other published studies listed in Table S2. Coordinates of PASs from these sources were individually converted to the aforementioned genome version for each species using the UCSC LiftOver tool and were then deduplicated. After that, we obtained unique known PASs for seven species: 921,664 in human, 256,331 in rhesus macaque, 580,462 in mouse, 331,743 in rat, 334,496 in zebrafish, 115,506 in fruit fly, and 46,439 in worm (Figure 1C), which are accessible at https://github.com/YangLab/SCAPTURE-v2.

These known PASs were then used to compare with those called by SCAPTURE (see below) from scRNA-seq datasets in this study. A SCAPTURE-identified PAS peak was labeled as “overlapping” PAS if its region (including 50 nt upstream and 25 nt downstream of the 3′ end) overlapped with a known PAS. All other peaks were then classified as “non-overlapping” PASs.

**Establishment of species-specific DeepPASS models and species-specific SCAPTURE v2**

Firstly, different strategies were applied to obtain positive PAS sites for species-specific DeepPASS model construction. In mouse, 122,882 stringent PAS sites from known PASs in GENCODE or those consistently present in all three of PolyA_DB3, PolyASite (v2.0), and PolyA-seq databases were obtained for the mouse-specific model development. In other non-human/mouse species, such as rhesus macaque, rat, zebrafish, worm and fruit fly, as their known PASs are much fewer than those in human or mouse, all known PASs, instead of intersected ones, were chosen for the species-specific model development (Figure 1C and Table S2).

As previously reported [9], these positive PAS sets of each species were respectively split into a model training set and an independent validation set with a 9:1 ratio. To simulate potential bias in peak-calling, we generated (1) positive sequences comprising unique 200-nucleotide regions centered on each polyadenylation site (PAS), with positions randomly shifted by a normal distribution (μ = 0, σ = 10 nucleotides), and (2) negative sequences with equal length were randomly selected from intergenic regions without known PASs. Of note, redundant sequences were removed before embedding for species-specific DeepPASS model training (batch size: 5000; epochs: 10). Early stopping was set with a patience of 10 rounds to terminate training process if no increased performance was observed. The model with best performance during training was kept for each species (Figure 1C**)**. As a result, all species-specific DeepPASS models were shown to achieve better performance on PAS calling with their corresponding species-specific DeepPASS models than those with the original human DeepPASS model (Figure 1C).

Interestingly, when using less stringent PAS sites identified in at least two of three aforementioned databases, or in GENCODE annotation for model development, the performance of trained models in human and mouse declined (data not shown). It thus indicated that training with high-confidence PAS sites was important for the model performance. In the future, when more reliable PASs are available, better species-specific models could be further developed, especially in those non-human/mouse species.

Finally, species-specific SCAPTURE v2 pipeline was developed with embedding species-specific DeepPASS models.

**PAS calling with SCAPTURE v2**

PAS calling was performed with SCAPTURE v2 pipeline, as described in previous study [9]. Briefly, we used annotation module (*scapture -m annotation*) to create reference files for PAS identification based on genome and gene annotations, and then made PAS calling from scRNA-seq datasets with PAScall module (*scapture -m PAScall -a SCAPTURE_annotation -g SCAPTURE_annotation.fa -b bam -o out_prefix --polyaDB SCAPTURE_annotation.pADB.txt*) in SCAPTURE v2 pipeline. In this PAS calling step, raw PAS peaks that were selected as “positive” by embedding species-specific DeepPASS models or overlapping with known PASs were classified as high-confidence PASs and kept for subsequent analyses.

**Integration of PASs identified from multiple samples**

Not surprisingly, PAS peak calling tended to shift a little bit from scRNA-seq samples across different tissues/studies, while they might indicate the same PAS sites (Figures S1B and Figure S1C). In this case, a two-step integration framework was implemented to improve reliability in PAS calling and subsequent comparison across different tissues/studies (Figure S1C). First, nearby peaks were merged if having at least 60% overlap in their summit-centered regions, ensuring that the merged loci capture the maximal signal of all nearby peaks. Next, DeepPASS-based scores were applied to prioritize PAS candidates with high-confidence sequence features, and the peak with the highest DeepPASS score was selected as the representative PAS for subsequent analyses. This strategy optimizes PAS detection within the current analytical framework by balancing peak signal robustness with sequence specificity across different tissues/studies.

**Feature analyses of polyadenylation signals around PASs**

Context sequences around SCAPTURE-identified PASs were extracted with bedtools to analyze the features of polyadenylation signals including hexamers AAUAAA and its close variants (AUUAAA, A[G/C]UAAA, AA[G/C]AAA), U/GU-rich elements and UGUA elements. Percentages of hexamers AAUAAA and its variants identified within the upstream 50 nt regions of all SCAPTURE-identified PASs were shown in Figure 2B. Distribution of hexamers AAUAAA and its variants, U/GU-rich elements and UGUA elements in 200 nt regions (±100nt) of 3' UTR PASs were profiled by MEME *CentriMo* (v 5.4.1) (parameter: *--verbosity 1 --norc*), indicated in Figure 4B.

**PAS validation with independent nanopore long-read sequencing datasets**

Nanopore long-read sequences containing both oligo(A) and adaptor sequences at their 3′ ends were selected for pileup, which determine their 3′ terminal alignment sites as potential nanopore-indicated PASs. Of note, only those without poly-A tracts (≥ 8 continuous A bases) in upstream 12nt regions were selected as high-confidence nanopore-indicated PASs for further comparison with SCAPTURE-identified PASs. If a SCAPTURE-identified PAS could overlap with a nanopore-indicated PAS site within its upstream 50nt to downstream 25nt region, this SCAPTURE-identified PAS was then classified as a validated one.

**Statistical analysis of PAS usage**

PAS count matrix was generated by *PASquant* module of SCAPTURE (*scapture -m PASquant -b BAM --celllist cell_annotation.list --pas SCAPTURE.PASquant.bed*) for each scRNA-seq sample, according to cell barcode files. CPMs (counts per million mapped reads) were used to demonstrate PAS expression levels, normalized from PAS counts.

Furthermore, we employed two metrics, *PAS proportion* and *PAS rate*, to evaluate the PAS usage level in PASSpedia.

The *PAS proportion* of one cell was calculated to indicate the percentage of a given PAS expression divided by total expression of all PASs at the same gene locus, according to previous publications [10,11]. For example, *dPAS proportion* was calculated using following formula:

$$\begin{aligned} dPAS proportion= \frac{{CPM}_{dPAS}}{\sum{CPM}_{{PAS}_{i}}} \# \end{aligned}$$

where *i* is any PAS of a given gene locus. Of note, for cross-species analysis of dPAS usage (Figure 5B), *dPAS proportion* of each cell type was calculated with mean CPM of dPASs for a given gene from all single cells belonging to the same cell type. This approach can effectively reduce the effect of dropouts in single-cell data and was applied in the comparison of PAS usage among cell types.

In addition, *PAS rate* could be also applied to represent PAS usage in single cell resolution according to our previous publication [9], defined by the log_2_ ratio of a given PAS (for example dPAS) to all the other PASs at a given gene locus:

$$\begin{aligned} dPAS rate= {log}_{2} \frac{{CPM}_{dPAS}+1}{\sum{CPM}_{rest}+1} \# \end{aligned}$$

To assess the global preference of dPAS (or pPAS) for each cell, we calculated the average of *PAS proportion* or *PAS rate* for dPAS (or pPAS) of all genes in each cell. The mean PAS usage level (*PAS proportion* or *PAS rate*) of all genes was used to present PAS performance of one cell then scaled by Z-score for all cells in a given sample and shown in PASSpedia “Analyze” module *via* UMAP plot or violin plot.

**Tissue specificity evaluation of PASs**

The *tau*(τ) index [12] was used to evaluate the tissue specificity of PASs according to the following equation:

$$\tau=\frac{\sum_{i=1}^{N} \left( 1-x_{i} \right)}{N-1}$$

where *N* is the number of analyzed tissues and $x_{i}$ is the PAS CPM normalized by the maximal value of PASs in *N* tissues.

**PAS sequence conservation across species**

PAS sequence conservation was analyzed according to previous studies [13,14]. Briefly, 24nt context sequences (±12nt) around 3′ ends of SCAPTURE-identified PASs were extracted and compared by UCSC LiftOver tool (-minMatch=0.1 -bedPlus=6) to identify conserved PASs between two species. PAS sequence conservation analyses were performed from each pair of human, mouse and zebrafish.

**Cell type classification**

Single cell datasets of eleven human tissues (bladder, brain, heart, kidney, intestine, muscle, liver, lung, pancreas, spleen and testis) from Tabula Sapiens [15], Guo et al. [16] and Bakken et al. [1], and matched mouse samples from Tabula Muris [17], Ernst et al. [18] and Bakken et al. [1] were selected for gene expression and PAS usage analyses. Cell type annotation was obtained from either original studies or further clustering by Seurat package with standard pipelines according to provided marker genes.

**Integrated analyses of human and mouse single-cell datasets**

Gene expression matrices of scRNA-seq and their corresponding cell annotation files were integrated for subsequent analyses. Briefly, orthologous gene annotation files of human and mouse from Ensembl database were screened by “ortholog_one2one” tag to obtain the one-to-one homologous gene list. A human-mouse co-expressed orthologous gene matrix was extracted from the original gene expression matrix from each scRNA-seq sample, labelled with same gene name symbols in both human and mouse gene expression matrices. Each human-mouse co-expressed orthologous gene expression matrix from every sample was normalized by SCTransform function of Seurat package with default parameters. After the human-mouse orthologous gene expression matrices initially merged (by the “merge” command), the top 2000 highly variable genes overlapped in all scRNA-seq samples were selected by SelectIntegrationFeatures function (object.list= c(seurat_list.m, seurat_list.h), nfeatures = 2000). These highly variable genes were used to perform dimensional reduction by RunPCA function of Seurat package.

RunHarmony function of harmony package was used to integrate different batches of scRNA-seq samples from human and mouse with parameter (*group.by.vars = c(“tech”), plot_convergence = T, reduction = “pca”, dims.use = 1:30, theta = c(0.5), lambda = c(0.5), max.iter.harmony = 10, max.iter.cluster = 20, epsilon.cluster = -Inf, epsilon.harmony = -Inf*), which was accomplished through the first 30 principal component vectors for PCA analysis.

The *k*-nearest neighbors graph (*k*-NNG) between cells was constructed by using the first 30 principal components through the FindNeighbors function of Seurat, followed by unsupervised cell clustering through the FindClusters function, with resolution between 0.1 and 0.5. UMAP visualization for dimensionality reduction was performed by RunUMAP function.

**Selection of consistent cell types in human and mouse samples**

To identify corresponding relation between human cell types and mouse cell types, we defined a *similarity index* (range from 0~1) to denote the cell type consistency between human and mouse samples. The higher value of the similarity score indicated the higher cell type consistency between human and mouse. The similarity index (SI) was calculated using the following formula:

$$\begin{aligned} SI(h, m)=\sum_{i}^{C} min\left( P_{i}^{h}, P_{i}^{m} \right)\# \end{aligned}$$

where *h* and *m* represent specific human and mouse cell types. *i* represents a cluster from the re-clustering of human and mouse cells and *C* is the set of all clusters. $P_{i}^{h}$(or $P_{i}^{m}$) is the proportion of cell type *h* (or *m*) classified into cluster *i*. The minimal one in $P_{i}^{h}$and $P_{i}^{m}$ was chosen for cluster *i* in *C* and all values were summed up as *SI*(*h*,*m*). For a given human cell type, SIs of all mouse cell types were computed, and the mouse cell type with the highest SI score was regarded as the consistent cell type to human.

During re-clustering, unsupervised cell clustering was performed with resolution between 0.1 to 0.5 for better effect. Of notes, different resolution was used in original cell type annotations of human and mouse, such as “T cells” annotated in human was annotated as “CD8^+^ T cells” and “CD4^+^ T cells” in mouse, which might cause “one-to-many” or “many-to-many” cell type correspondence. This condition was solved by splitting one cell type into more types or combining multiple cell types into one type according to overlap ratio (0.5 in this study). Of note, any cell clusters that have fewer than 30 cells were removed to ensure statistical robustness and biological relevance.

**Dimensionality reduction analysis of cellular gene expression and PAS usage**

The multi-PAS genes that expressed (CPM ≥ 5) in more than half cell types were kept for the following analysis. The cell-type level dPAS proportion (described in “Statistical analysis of PAS usage”) was calculated by custom R script for multi-PAS genes with CPM ≥ 5. The genes with standard deviation of gene expression higher than 1 were identified as variable expression genes, while those with standard deviation of dPAS proportion higher than 0.2 were filtered as variable PAS usage genes. Subsequently, the scaled variable features were calculated to perform dimensionality reduction analysis using R package UMAP (v 0.2.8.0).

**References**

[1] Bakken TE, Jorstad NL, Hu QW, Lake BB, Tian W, Kalmbach BE, et al. Comparative cellular analysis of motor cortex in human, marmoset and mouse. Nature 2021;598:111–9.

[2] Zheng GXY, Terry JM, Belgrader P, Ryvkin P, Bent ZW, Wilson R, et al. Massively parallel digital transcriptional profiling of single cells. Nat Commun 2017;8:14049.

[3] Stuart T, Butler A, Hoffman P, Hafemeister C, Papalexi E, Mauck WM, et al. Comprehensive Integration of Single-Cell Data. Cell 2019;177:1888–902.

[4] You LM, Wu JX, Feng YC, Fu YG, Guo YN, Long LY, et al. APASdb: a database describing alternative poly(A) sites and selection of heterogeneous cleavage sites downstream of poly(A) signals. Nucleic Acids Res 2015;43:D59–D67.

[5] Jin WW, Zhu QZ, Yang YB, Yang WQ, Wang DY, Yang JJ, et al. Animal-APAdb: a comprehensive animal alternative polyadenylation database. Nucleic Acids Res 2021;49:D47–D54.

[6] Herrmann CJ, Schmidt R, Kanitz A, Artimo P, Gruber AJ, Zavolan M. PolyASite 2.0: a consolidated atlas of polyadenylation sites from 3′ end sequencing. Nucleic Acids Res 2020;48:D174–D9.

[7] Derti A, Garrett-Engele P, Macisaac KD, Stevens RC, Sriram S, Chen R, et al. A quantitative atlas of polyadenylation in five mammals. Genome Res 2012;22:1173–83.

[8] Wang RJ, Nambiar R, Zheng DH, Tian B. PolyA_DB 3 catalogs cleavage and polyadenylation sites identified by deep sequencing in multiple genomes. Nucleic Acids Res 2018;46:D315–D9.

[9] Li GW, Nan F, Yuan GH, Liu CX, Liu X, Chen LL, et al. SCAPTURE: a deep learning-embedded pipeline that captures polyadenylation information from 3' tag-based RNA-seq of single cells. Genome Biol 2021;22:221.

[10] Lee S, Chen YC, Consortium FCA, Gillen AE, Taliaferro JM, Deplancke B, et al. Diverse cell-specific patterns of alternative polyadenylation in Drosophila. Nat Commun 2022;13:5372.

[11] Zingone A, Sinha S, Ante M, Nguyen C, Daujotyte D, Bowman ED, et al. A comprehensive map of alternative polyadenylation in African American and European American lung cancer patients. Nat Commun 2021;12:5605.

[12] Yanai I, Benjamin H, Shmoish M, Chalifa-Caspi V, Shklar M, Ophir R, et al. Genome-wide midrange transcription profiles reveal expression level relationships in human tissue specification. Bioinformatics 2005;21:650–9.

[13] Wang RJ, Zheng DH, Yehia G, Tian B. A compendium of conserved cleavage and polyadenylation events in mammalian genes. Genome Res 2018;28:1427–41.

[14] Lee JY, Ji Z, Tian B. Phylogenetic analysis of mRNA polyadenylation sites reveals a role of transposable elements in evolution of the 3′-end of genes. Nucleic Acids Res 2008;36:5581–90.

[15] Jones RC, Karkanias J, Krasnow MA, Pisco AO, Quake SR, Salzman J, et al. The Tabula Sapiens: A multiple-organ, single-cell transcriptomic atlas of humans. Science 2022;376:eabl4896.

[16] Guo JT, Grow EJ, Mlcochova H, Maher GJ, Lindskog C, Nie XC, et al. The adult human testis transcriptional cell atlas. Cell Res 2018;28:114–57.

[17] Almanzar N, Antony J, Baghel AS, Bakerman I, Bansal I, Barres BA, et al. A single-cell transcriptomic atlas characterizes ageing tissues in the mouse. Nature 2020;583:590–5.

[18] Ernst C, Eling N, Martinez-Jimenez CP, Marioni JC, Odom DT. Staged developmental mapping and X chromosome transcriptional dynamics during mouse spermatogenesis. Nat Commun 2019;10:1251.
