# Supplementary material for: PASSpedia: A Polyadenylation Site Database Across Different Species at Single‐cell Resolution
Source: Genomics Proteomics Bioinformatics. 2025 Sep 23;24(2):qzaf089. doi: 10.1093/gpbjnl/qzaf089 (PMC13309242; doi:10.1093/gpbjnl/qzaf089)
Supplement: qzaf089_Supplementary_Data [file qzaf089_supplementary_data.zip › qzaf089 Supplementary data-new.zip!/Supplementary material captions.docx]

**Supplementary material**

**File S1 Supplementary method**

**Figure S1 Development of species-specific SCAPTURE v2 pipelines embedded with species-specific DeepPASS models**

**A.** Summary of collected single-cell data for PAS identification. **B.** Stepwise SCAPTURE v2 pipeline for PAS analysis. **C.** Schematic of the modified *PASmerge* module in SCAPTURE v2.

**Figure S2 Nucleotide distribution of SCAPTURE-identified PASs across seven species**

Distribution of four different nucleotides (AUGC) among SCAPTURE-identified exonic, intronic, and 3'-extended PASs within 200nt (±100nt) regions was compared with that among known PASs. All SCAPTURE-identified PASs were divided into two subgroups: ones overlapped with known PASs and the others non-overlapped with known PASs.

**Figure S3 Analyses of SCAPTURE-identified exonic PASs**

**A.** Comparison of SCAPTURE-identified exonic PASs with known PASs across seven species. SCAPTURE-identified exonic PASs that were overlapped or non-overlapped with known exonic PASs were indicated in purple or pink, respectively. Only a subset of SCAPTURE-identified human exonic PASs, which were detected from 3' tag-based scRNA-seq of ten human tissue samples with corresponding Nanopore long-read sequencing data, were retained for comparison in B, C and D. **B.** Expression comparison of overlapped and non-overlapped PASs in ten human tissues. Statistical significance was assessed with Student’s *t*-test. n.s., non-significant. *P* ≥ 0.5; **P* < 0.05; ***P* < 0.01; ****P* < 0.001. **C.** Numbers of SCAPTURE-identified exonic PASs that could be validated (blue) or non-validated (grey) by nanopore long-read sequencing data from overlapped (left) or non-overlapped (right) exonic PASs. **D.** Numbers of highly expressed SCAPTURE-identified exonic PASs that could be validated or non-validated by nanopore long-read sequencing data. Highly expressed SCAPTURE-identified exonic PASs were selected with CPM ≥ 5 in single-cell data. Left, overlapped exonic PASs with CPM ≥ 5. Right, non-overlapped exonic PASs with CPM ≥ 5. **C.** and **D.** Percentages of validated PASs in all ten human tissue were labeled next to tissue names.

**Figure S4 Analyses of SCAPTURE-identified intronic PASs**

**A.** Percentages of internal poly-A tracts (≥ 8 continuous A bases) within 600nt (±300nt) regions around exonic or intronic PASs. **B.** and **C.** Mean expression (CPM, B) and specificity (*Tau* index, C) of exonic and intronic PASs.

**Figure S5 Analyses of SCAPTURE-identified intergenic (3**'**-extended 2000nt) PASs**

**A.** Percentages of internal poly-A tracts (≥ 8 continuous A bases) within 600 nt (±300 nt) regions around exonic or 3'-extended PASs. **B.** and **C.** Mean expression (CPM, B) and specificity (*Tau* index, C) of exonic and 3'-extended PASs.

**Figure S6 Clusters of gene expression and dPAS usage in eleven human tissues**

**A.** Sankey diagram showing the flow of 80 human cell types between 11 tissues and 9 cell classes, indecating the composition of cell types in each tissue and cell class. **B.** Selection of 5942 highly expressed (CPM ≥ 5) multi-PAS mRNA genes. **C.** UMAP plot showing the dimensionality reduction results of 80 human cell types based on gene expression level (left) and dPAS proportion (right).

**Figure S7 Clusters of gene expression and dPAS usage in eleven mouse tissues**

**A.** Sankey diagram showing the flow of 80 mouse cell types between 11 tissues and 9 cell classes, indecating the composition of cell types in each tissue and cell class. **B.** Selection of 5119 highly expressed (CPM ≥ 5) multi-PAS genes. **C.** UMAP plot showing the dimensionality reduction results of 80 mouse cell types based on gene expression level (left) and dPAS proportion (right).

**Table S1 List of 3' tag-based scRNA-seq datasets of seven species used in this study**

**Table S2 All annotation used in this study**

Sheet1, known PASs of seven species from various available databases. Sheet2, genomic references and gene annotations for the development of species-specific SCAPTURE v2 pipeline.

**Table S3 All SCPATURE-identified exonic PASs**

Of note, other layers of information, such as “3' UTR” PASs and “overlapped/non-overlapped with known PASs”, were also labelled. Human PASs that could be validated with nanopore long-read sequencing data were additionally labeled with “nanopore-seq validated”.

**Table S4 All SCPATURE-identified intronic PASs**

Each PAS site labeled with tag “with/without poly-A tracts (≥ 8 continuous A bases)”.

**Table S5 All SCPATURE-identified 3'-extended PASs**

Each PAS site labeled with tag “with/without poly-A tracts (≥ 8 continuous A bases)”.

**Table S6 List of 80 cell types spanning 9 cell classes from 11 tissues**

**Table S7 List of multi-3'UTR-PAS mRNA genes of human and mouse**

**Table S8 List of 4351 human-mouse orthologous genes with conserved dPASs**
